# Glycomics of cervicovaginal fluid from women at risk of preterm birth reveals immuno-regulatory epitopes that are hallmarks of cancer and viral glycosylation

**DOI:** 10.1038/s41598-024-71950-x

**Published:** 2024-09-06

**Authors:** Gang Wu, Paola Grassi, Belen Gimeno Molina, David A. MacIntyre, Lynne Sykes, Phillip R. Bennett, Anne Dell, Stuart M. Haslam

**Affiliations:** 1 Department of Life Sciences, Imperial College London, London, UK; 2 March of Dimes Prematurity Research Centre at Imperial College London, London, UK; 3 Institute of Reproductive & Developmental Biology, Imperial College London, Hammersmith Hospital Campus, Du Cane Road, London, UK; 4 The Parasol Foundation Centre for Women’s Health and Cancer Research, St Mary’s Hospital, London, W1 2NY UK

**Keywords:** Biochemistry, Glycobiology, Glycomics

## Abstract

During pregnancy the immune system needs to maintain immune tolerance of the foetus while also responding to infection, which can cause premature activation of the inflammatory pathways leading to the onset of labour and preterm birth. The vaginal microbiome is an important modifier of preterm birth risk, with *Lactobacillus* dominance during pregnancy associated with term delivery while high microbial diversity is associated with an increased risk of preterm birth. Glycans on glycoproteins along the lower female reproductive tract are fundamental to microbiota-host interactions and the mediation of inflammatory responses. However, the specific glycan epitopes involved in these processes are not well understood. To address this, we conducted glycomic analyses of cervicovaginal fluid (CVF) from 36 pregnant women at high risk of preterm birth and 4 non-pregnant women. Our analysis of N- and O-glycans revealed a rich CVF glycome. While O-glycans were shown to be the main carriers of ABO blood group epitopes, the main features of N-glycans were the presence of abundant paucimannose and high mannose glycans, and a remarkable diversity of complex bi-, tri-, and tetra-antennary glycans decorated with fucose and sialic acid. We identified immuno-regulatory epitopes, such as Lewis antigens, and found that fucosylation was negatively correlated to pro-inflammatory factors, such as IL-1β, MMP-8, C3a and C5a, while glycans with only sialylated antennae were mainly positively correlated to those. Similarly, paucimannose glycans showed a positive correlation to pro-inflammatory factors. We revealed a high abundance of glycans which have previously been identified as hallmarks of cancer and viral glycosylation, such as Man8 and Man9 high mannose glycans. Although each pregnant woman had a unique glycomic profile, longitudinal studies showed that the main glycosylation features were consistent throughout pregnancy in women who delivered at term, whereas women who experienced extreme preterm birth exhibited sharp changes in the CVF glycome shortly before delivery. These findings shed light on the processes underlying the role of glycosylation in maintaining a healthy vaginal microbiome and associated host immune responses. In addition, these discoveries facilitate our understanding of the lower female reproductive tract which has broad implications for women’s health.

## Introduction

Human pregnancy is a balancing act: the immune system is carefully steered to maintain an immune tolerance of the semi-allogeneic foetus^1^, whilst maintaining capacity to respond to pathogenic infection. During embryo implantation and placentation, the female reproductive tract is reflective of a pro-inflammatory state which subsequently transitions to an anti-inflammatory environment to facilitate foetal growth and tolerance of paternal antigens^2^. This is maintained in healthy pregnancies until late gestation and the onset of parturition. Labour is a pro-inflammatory state, in which leukocytes (macrophages and neutrophils in particular) infiltrate the human myometrium, foetal membranes and the cervix. Proinflammatory cytokines such as IL-1β, IL-6, IL-8 and TNF-α are released, contributing to the activation of uterine contractions, rupturing of the foetal membranes, cervical remodelling and dilation.^3–5^ (Supplementary Fig. S1a).

Untimely activation of inflammatory pathways in gestational tissues can lead to preterm birth, defined as delivery before 37 weeks of completed gestation. An estimated 40% of all preterm birth cases are linked with an infectious aetiology^6^, with pathogen colonisation of the cervicovaginal niche followed by ascension towards the maternal-foetal interface considered as a major route of infection^7,8^. Displacement of commensal *Lactobacillus* species from the vagina during pregnancy is associated with an increase in pro-inflammatory cytokines, early cervical remodelling and increased risk of spontaneous preterm birth^7,9,10^. This response is often characterised by recruitment of immune cells, in particular neutrophils^11^, to the foetal membranes and release of immune mediators at the cervicovaginal interface^12^. Consistent with these observations, the vaginal microbiome is now considered an important modifier of preterm birth risk^13–15^. Compositionally, the vaginal microbiome can be classified into five community state types (CSTs), of which four are primarily composed of a single dominant species of *Lactobacillus* (*L. crispatus* (CST-I), *L. gasseri* (CST-II), *L. iners* (CST-III) and *L. jensenii* (CST-V) respectively), and one diverse community (CST-IV) typically comprising *Gardnerella*, *Prevotella*, *Sneathia*, *Atopobium*, *Molibuncus*, *Clostridium*, *Corynebacterium*, *Staphylococcus*, *Streptococcus*, *Enterococcus*, and *Mycoplasma*^7,16^. CST I can be further divided into CST I-A and I-B based on the degree of *L. crispatus* dominance, and CST III can be divided into CST III-A and III-B reflective of the degree of *L. iners* dominance. CST IV can be further classified as CST IV-A, with a moderate relative abundance *of Gardnerella*, and CST IV-B reflective of a high relative abundance of *Gardnerella*. *L. crispatus* dominance (CST I) during pregnancy associates with term delivery, whereas *L. iners* (CSTIII) and high diversity (CST IV) associates with an increased risk of preterm births^17–19^.

The mechanisms underpinning compositional changes in the vaginal microbiome and subsequent host immune responses remain poorly characterised. However, many proteins with putative roles in mediating microbiota-host responses within the human female reproductive tract share several features including being heavily glycosylated with a rich repertoire of glycans^20–22^. These include important functional sequences (glycotopes), such as Lewis antigens, SLewis antigens, ABO blood groups, and poly N-acetyllactosamine (polyLacNAc) antennae (structures shown in Supplementary Fig. S1), which can serve as ligands for glycan binding proteins (GBPs; also called lectins) from both the immune system and the microbes^23^ (Supplementary Fig. S1 b–d). Glycan-lectin engagement is predicted to regulate key aspects of cell–cell recognition, adhesion, signalling and host–pathogen/host commensal interactions during human reproduction but the specific glycan epitopes involved have been challenging to identify^24^. To begin to address this, we recently described procedures and workflows that allow glycomic analysis of cervicovaginal fluid (CVF) during pregnancy^25^. In this study of a small cohort of 10 donors, high sensitivity mass spectrometric methods revealed a rich and complex CVF N-glycome, characterised by abundant paucimannose and high mannose glycans, as well as a remarkable diversity of complex bi-, tri- and tetra-antennary glycans whose antennae were extensively decorated with fucose and sialic acid^25^. A substantial portion of the complex-type N-glycome carried extended polylacNAc antennae. Quantitative profiling of fucosylation and sialylation of the smaller complex glycans provided the first physicochemical evidence that expression of specific glycan epitopes is related to the microbial community status and immune activity in the cervicovaginal region. In the present study, we substantially expanded the scope of our investigations to describe global and temporal N- and O-glycomics of CVF samples (*n* = 60) collected from 36 pregnant women at high risk of preterm birth and 4 non-pregnant women. Of the pregnant women, 15 delivered preterm and 21 at term (Supplementary Table S1). Our expanded glycomics analyses included identification of terminal glycotopes displayed on extended polylacNAc antennae, which are expected to act as more accessible ligands for lectins compared to counterparts on non-extended antennae. Many CVF samples were remarkably rich in glycan epitopes widely considered to be unique hallmarks of cancer and viral glycosylation. Complementary functional analyses revealed quantitative differences in glycan epitope expression associated with bacterial composition and concentrations of cytokines, matrix metalloproteinases (MMP) and complement proteins. Longitudinal glycomic studies of two women who delivered extreme preterm showed substantial changes in expression of immune-active glycan epitopes shortly before delivery. In contrast, glycomes remained stable into the third trimester for women who delivered at term. We also present an analysis strategy designed to address issues of overlapped isotopic peaks from multiple glycans commonly found in the high mass range of the complex CVF N-glycome. This approach facilitated accurate high-speed data annotation and quantitation enabling the construction of N-glycan, O-glycan and glycotope libraries, which are provided as open access repositories.

## Results

### Patient cohort and overview of CVF glycomic characterisation

A total of 60 CVF samples were collected from 36 pregnant donors at high risk of preterm birth between 10 and 34 weeks of gestation. Of these, 15 women subsequently delivered preterm (< 37 weeks gestation) and 21 delivered at term (≥ 37 weeks). Details of the sample cohort and gestational age at sampling is provided in Supplementary Fig S2. Of these women, 15 donors were blood group O, 13 were blood group A and 12 were blood group B. CVF samples were also collected from four non-pregnant donors to enable comparative analyses. All CVF samples were subjected to MALDI-TOF/TOF N-glycomic analysis. In addition, O-glycomics and profiling of glycotopes on extended antennae (derived from both N- and O-glycans) were performed on samples that were available in sufficient quantities for multiple experiments. The list of glycomic datasets obtained for each sample is provided in Supplementary Table S2 and the online repository (https://github.com/gw110/Glycomics-of-cervicovaginal-fluid-from-women-at-risk-of-preterm-birth.git).

### Annotation and quantitation of N-glycans in overlapped mass spectrometry peaks

Due to the complexity of glycosylation in the CVF samples, overlapping molecular ion isotope clusters were commonly observed, especially in the high m/z region of the N-glycan spectra. An in-house bioinformatic tool was developed to accurately de-isotope overlapped peaks for accurate glycan assignment. A multinomial distribution model was first trained using standard permethylated glycan data and their molecular formulae. The model was then tested on poly hexoses and different types of N-glycans (Supplementary Fig. S1 a–b), both of which showed accurate prediction of mono-isotopic peak patterns. The optimized model was then used for deisotoping of an overlapped mono-isotopic peak cluster from a blood group A sample (Supplementary Fig. S3c). The deisotoped results showed an R-squared value as high as 0.99 for the peak cluster, and lack of a theoretical group B glycan at m/z 3955.0. Isotopic peak matchings for all glycomic data are freely accessible (https://github.com/gw110/Glycomics-of-cervicovaginal-fluid-from-women-at-risk-of-preterm-birth.git), accompanied by .csv tables summarising identifications of glycans from each CVF sample, including the sample name, sample collection time, mass, intensity, monosaccharide composition, and quality of monoisotopic peak pattern fit by R-squared values.

### Identification of a variety of distinctive N-glycan profile patterns in pregnant CVF

**Fig. 1:**
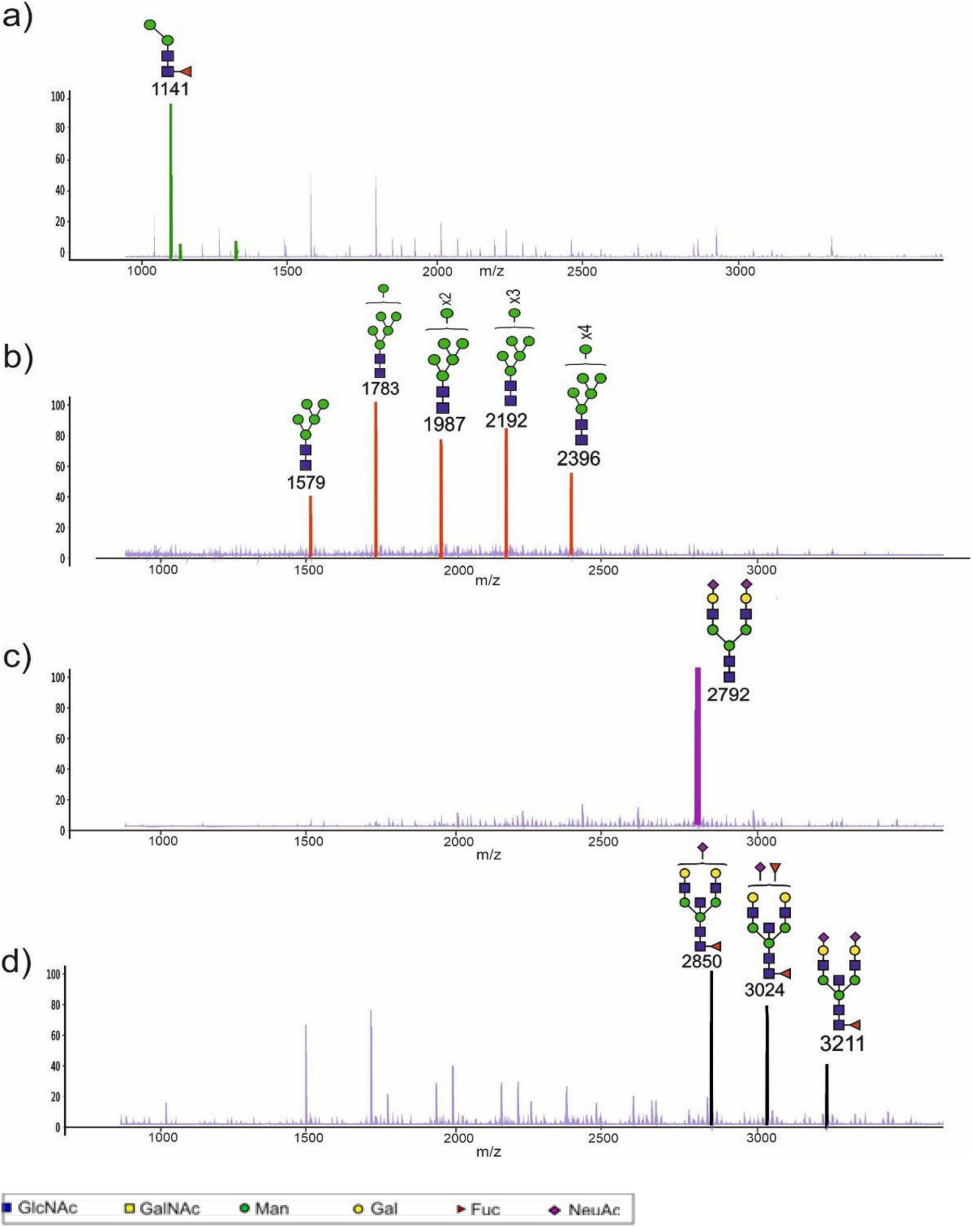
Representative MALDI-TOF mass spectra of 4 global N-glycan profile patterns. (**a**) Paucimannose dominated spectrum, from a blood group O donor with CST III-A who delivered at term. CVF sample was collected at 23 weeks and 6 days (23w6d) of gestation. Peaks highlighted in green correspond to paucimannose glycan structures, (**b**) high mannose dominated spectrum, from a blood group B donor with CST II who delivered at term. CVF sample was collected at 14w4d. Peaks highlighted in orange correspond to high mannose glycan structures, (**c**) fully sialylated non-fucosylated dominated spectrum, from a blood group B donor with CST I-A who delivered preterm. CVF sample was collected at 20w3d. The peak highlighted in purple corresponds to a sialylated non fucosylated structure, (**d**) bisected dominated spectrum, from a blood group B donor with CST I-A who delivered at term. CVF sample was collected at 22w5d. Peaks highlighted in black correspond to bisected glycan structures, confirmed by GC–MS linkage analysis. Assignments are based on composition, tandem MS and knowledge of biosynthetic pathways. All molecular ions are [M + Na]^+^

Detailed profiling of N-glycans of the 56 CVF samples from pregnant donors led to the discovery of unique global N-glycan profile patterns for each donor. Notably, complex-type glycans highly decorated with fucose and sialic acid on extended polylacNAc antennae were common to all the CVF samples. Nonetheless, samples could be categorised into one of six families, based on distinctive glycan signatures in the low to mid mass range of the N-glycomic spectra (https://github.com/gw110/Glycomics-of-cervicovaginal-fluid-from-women-at-risk-of-preterm-birth.git). Four of the families are illustrated in Fig. 1 and hierarchical clustering analysis of all CVF samples based on dominant glycans is shown in Supplementary Fig. S4. They are: (a) paucimannose dominant with the highest intensity peak at m/z 1141.5 corresponding to Fuc_1_Man_2_GlcNAc_2_ (Fig. 1a *n* = 10); (b) high mannose dominant, characterised by unusually high levels of Man_8_GlcNAc_2_ and Man_9_GlcNAc_2_ (Fig. 1b, *n* = 19); (c) spectra dominated by a single biantennary di-sialylated glycan without core fucose (m/z 2792.4, NeuAc_2_Gal_2_Man_3_GlcNAc_4_; Fig. 1c, *n* = 6); (d) spectra characterised by a high abundance of sialylated bisected glycans carrying one or 2 fucoses (at m/z 2850.4, 3024.5 and 3211.6, NeuAc_1-2_Fuc_1-2_Gal_2_Man_3_GlcNAc_5_) (Fig. 1d, *n* = 4). Linkage analysis showed a high level of 3,4,6-Man which confirms the presence of bisected glycan structures (Supplementary Table S3) The other two families exhibit combinations of (a-d). Thus family (e) has a strong signal at m/z 2792.4 together with abundant sialylated bisected glycans (mixture of c and d, *n* = 2), and family (f) displays characteristics of all families (a-d) with no clear dominance of any specific group of glycans (*n* = 19).

### Structural profiling of O-glycans

**Fig. 2:**
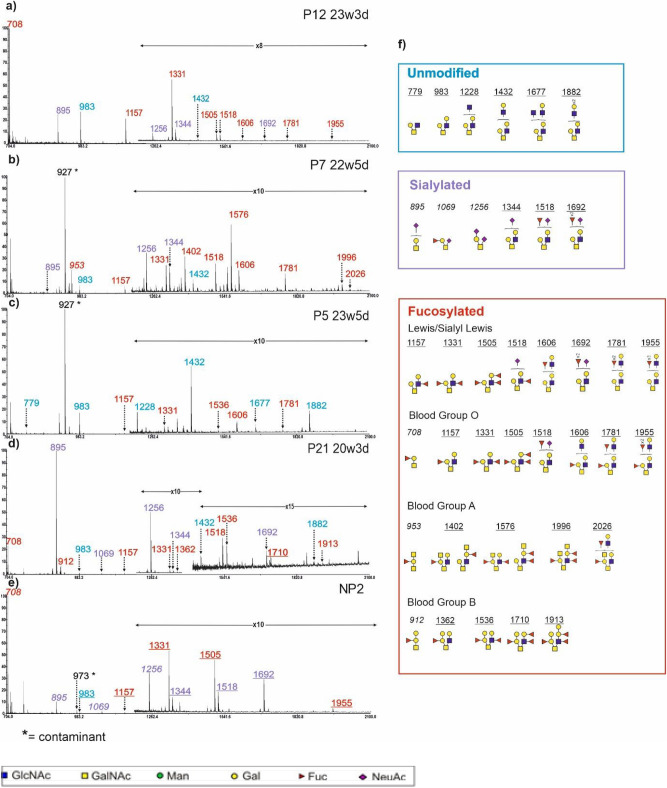
MALDI-TOF mass spectra (m/z 500–2000) of CVF O-glycans. (**a**) A blood group O donor with CST I-B who delivered at term; CVF was collected at 23 weeks and 3 days (23w3d) of gestation; (**b**) a blood group A donor with CST-II who delivered at term; CVF was collected at 22w5d; (**c**) a blood group B donor with CST IV-B who delivered preterm; CVF was collected at 23w5d; (**d**) a blood group B donor with CST I-A who delivered preterm; CVF was collected at 20w3d; (**e**) a blood group A non-pregnant donor. The O-glycans from CVF were released by reductive elimination and permethylated prior to MALDI-TOF and TOF-TOF profiling. Each spectrum is shown in a single panel, and all data are normalized to the most abundant component, which is designated as 100%. For clarity, a zoomed in panel is inserted for m/z above 900. Colour coding has been used to distinguish families of glycans: unmodified O-glycans are flagged as blue, peaks labelled in purple show sialylated O-glycans, while peaks labelled in red are fucosylated O-glycans. Structures of the colour coded peaks are displayed in the corresponding coloured rectangles in panel (**f**). Main structures are depicted. Assignments are based on composition, tandem MS and knowledge of biosynthetic pathways. All molecular ions are [M + Na]^+^. * Indicates non-glycan contaminant.

O-glycan data were obtained from 42 CVF samples (Supplementary Table S2). Both Core 1 and Core 2 structures were observed, as exemplified in Fig. 2. Unmodified Core 1 was identified at m/z 534 (GalGalNAc), while sialylated Core 1 structures were identified at m/z 895 (NeuAcGalGalNAc) and m/z 1256 (NeuAc_2_GalGalNAc). Fucosylated Core 1 carrying blood group epitopes H, A and B were identified at m/z 708 (FucGalGalNAc), 953 (FucGalGalNAc_2_) and 912 (FucGal_2_GalNAc) respectively (Fig. 2). A Core 1 structure modified by both fucose and sialic acid was detected at m/z 1069 (NeuAcFucGalGalNAc). In a small portion of samples, we identified structural isomers corresponding to elongated Core 1 structures, with the 3-linked arm modified by the addition of Gal, GlcNAc (eg. m/z 1432), Fuc and NeuAc residues (eg. m/z 1344). Most peaks with extended antennae were identified as Core2 O-glycans, with either or both the 3- and 6-linked antennae elongated by the addition of 1 to 4 LacNAc units (GalGlcNAc) and capped by a combination of up to 4 fucose and 2 sialic acid residues.

Several glycan epitopes of functional significance were found on O-glycans, including Lewis and SLewis epitopes. However, the most abundant epitopes identified on the O-glycans were the ABH blood group antigens, in contrast to the N-glycans where ABH blood group epitopes were only present on minor glycans. MS/MS analyses revealed different structural isomers carrying either Blood group H epitope in Blood Group O individuals or Lewis epitope in non-blood group O individuals (eg. m/z 1157). Interestingly, the blood group H epitope on the core 1 structure at m/z 708 is observed not only in Blood Group O donors but also in Blood Group A and B donors. Another interesting observation is the presence of internally fucosylated antennae carrying terminal blood group epitopes on several O-glycans (eg. m/z 1710, 1913).

### Structural profiling of N- and O- glycan derived glycotopes on extended antennae

**Fig. 3:**
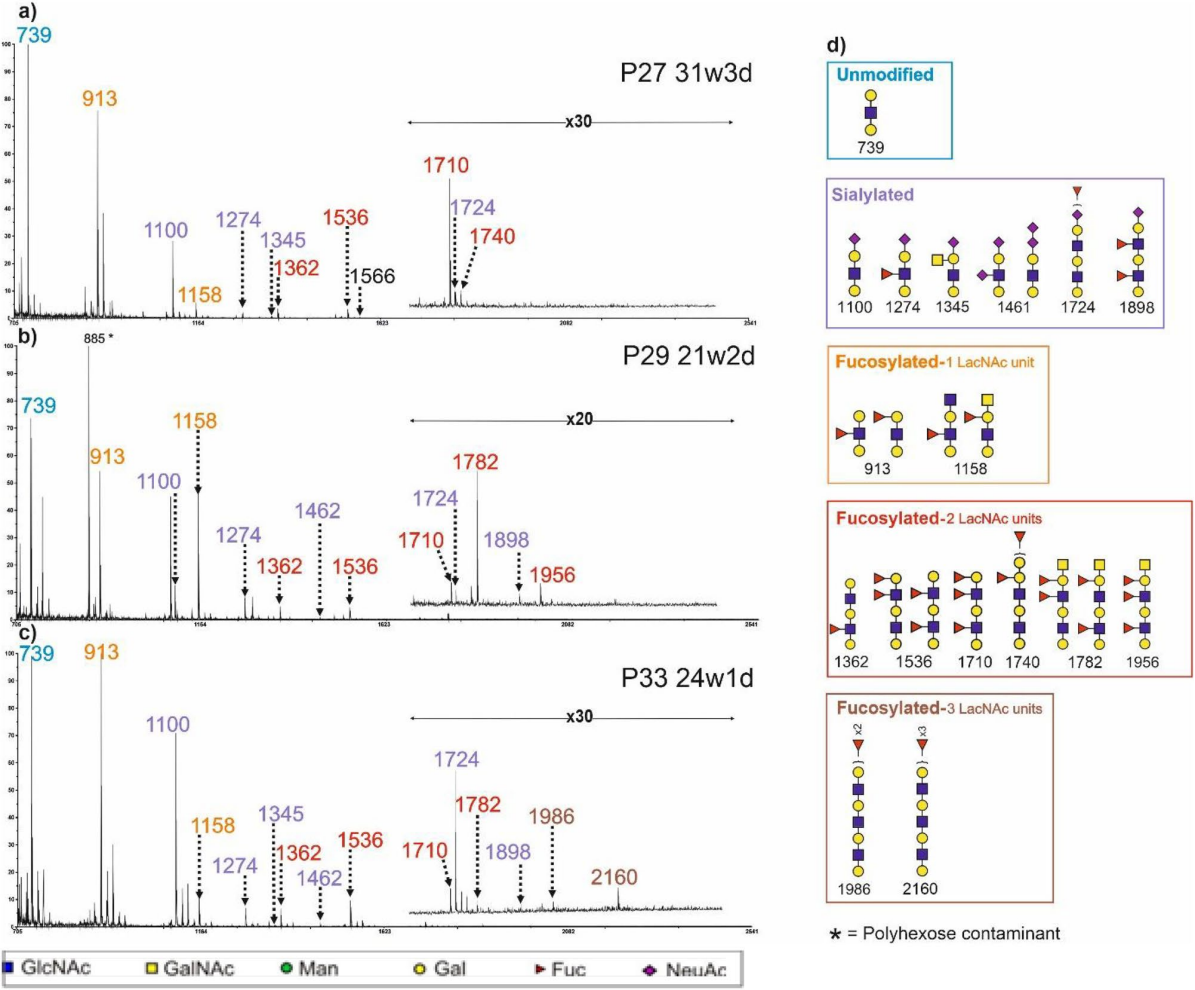
MALDI-TOF mass spectra (m/z 700–2500) of glycotopes isolated form CVF of: (**a**) a blood group B donor with CST I-B who delivered preterm. CVF was collected at 31 weeks and 3 days (31w3d) of gestation; (**b**) a blood group A donor with CST I-A who delivered at term. CVF was collected at 21w2d; (**c**) a blood group O donor with CST III-A who delivered at term. CVF was collected at 24w1d ; Glycotopes were released by endo-beta-galactose digestion and were deutero-reduced and permethylated prior to MALDI-TOF and TOF-TOF profiling. Each spectrum is shown in a single panel, and all data are normalized to the most abundant component, which is designated as 100%. For clarity, a zoomed in panel is inserted for m/z above 1600. Colour coding has been used to distinguish families of glycotopes: unmodified glycotopes are flagged as blue, peaks labelled in purple show sialylated glycotopes, peaks labelled in orange are fucosylated glycotopes with one LacNAc unit, peaks labelled in red are fucosylated glycotopes with two LacNAc units and peaks labelled in brown are fucosylated glycotopes with three LacNAc units. Structures of the colour coded peaks are displayed in the corresponding coloured rectangles in panel (**d**). Main structures are depicted. Assignments are based on composition, MS/MS and knowledge of biosynthetic pathways. All molecular ions are [M + Na]^+^.

Glycotope analyses were performed on 37 CVF samples by incubating glycopeptides with endo-β-galactosidase, which cleaves internal β1-4 galactose linkages in unbranched, repeating poly-N-acetyllactosamine (GlcNAc-β1-3Gal-β1-4)_n_ structures of both N- and O-glycans (Supplementary Fig S29, Supplementary Table S2). This included sample P34, whose preliminary glycan epitope analysis results were published in our earlier pilot study^25^. MALDI-MS spectra of glycotopes identified in three representative CVF samples are displayed in Fig. 3. The MS spectra are dominated by an unmodified tri-saccharide structure at m/z 739 corresponding to the sequence GalGlcNAcGal (the terminal cleavage product of endo-β-galactosidase digestion of uncapped polylacNAc antennae), and its fucosylated and sialylated variants at m/z 913 and 1100, respectively. After MS/MS analysis, the peak at m/z 913 was identified as a mixture of Lewis and Blood Group H epitopes. In blood group A donors, glycotopes carrying blood group epitopes were also identified at m/z 1158, 1782, 1956, 2405 and 2579 (Fuc_1-5_Gal_2-3_HexNAc_2-3_); glycotopes carrying blood group B epitopes were identified in blood group B donors at m/z 1566, 1740 and 1914 (Fuc_1-3_Gal_4_GlcNAc_2_) (Fig. 3). Several structures carrying multiple LacNAc units, multiple fucose residues and blood group epitopes were identified in the higher mass range, including structures carrying the Blood Group A epitope at m/z 2405, 2579, 3203 and 3228 which correspond to Fuc_3-5_Gal_3-5_HexNAc_3-4_, and structures carrying the Blood Group B epitope at m/z 2987, 3161 and 3610, which correspond to Fuc_4-5_Gal_6-7_GlcNAc_4-5_.

MS/MS analysis revealed the presence of the SdA epitope (NeuAcGal_2_GalNAcGlcNAc, m/z 1345), which was only identified on glycotopes but not on intact O- or N-glycans in CVF samples (Supplementary Fig. S5a). A di-sialylated glycotope at m/z 1461 (NeuAc_2_Gal_2_GlcNAc) was identified and characterized by MS/MS as a mixture of two structural isomers, one of which was derived from an extended antenna capped by a NeuAc-NeuAc moiety, whilst the other was derived from an extended antenna capped with a single NeuAc whilst the second NeuAc was linked to the GlcNAc residue (Supplementary Fig. S5b). Our observation of these glycotopes in this analysis, but not the intact O- or N-glycan analysis, indicates that they are present as very minor species. Several sialylated glycotopes were characterised: antennae with a backbone of 1 to 6 LacNAc units, combined with fucose to give SLewis X epitopes, spanning from smaller glycotopes at m/z 1274 (NeuAcFucGal_2_GlcNAc) and 1898 (NeuAcFuc_2_Gal_3_GlcNAc_2_) to larger glycotopes with m/z up to 4391 (NeuAcFuc_6_Gal_7_HexNAc_6_). MS/MS analysis of the structure at m/z 1724 (NeuAcFucGal_3_GlcNAc_2_) revealed that its fucose residue was on the internal LacNAc and not on the terminal LacNAc in most samples analysed by MS/MS (Supplementary Fig. S5c).

### Correlation of fucosylation and sialylation with inflammatory status

**Fig. 4:**
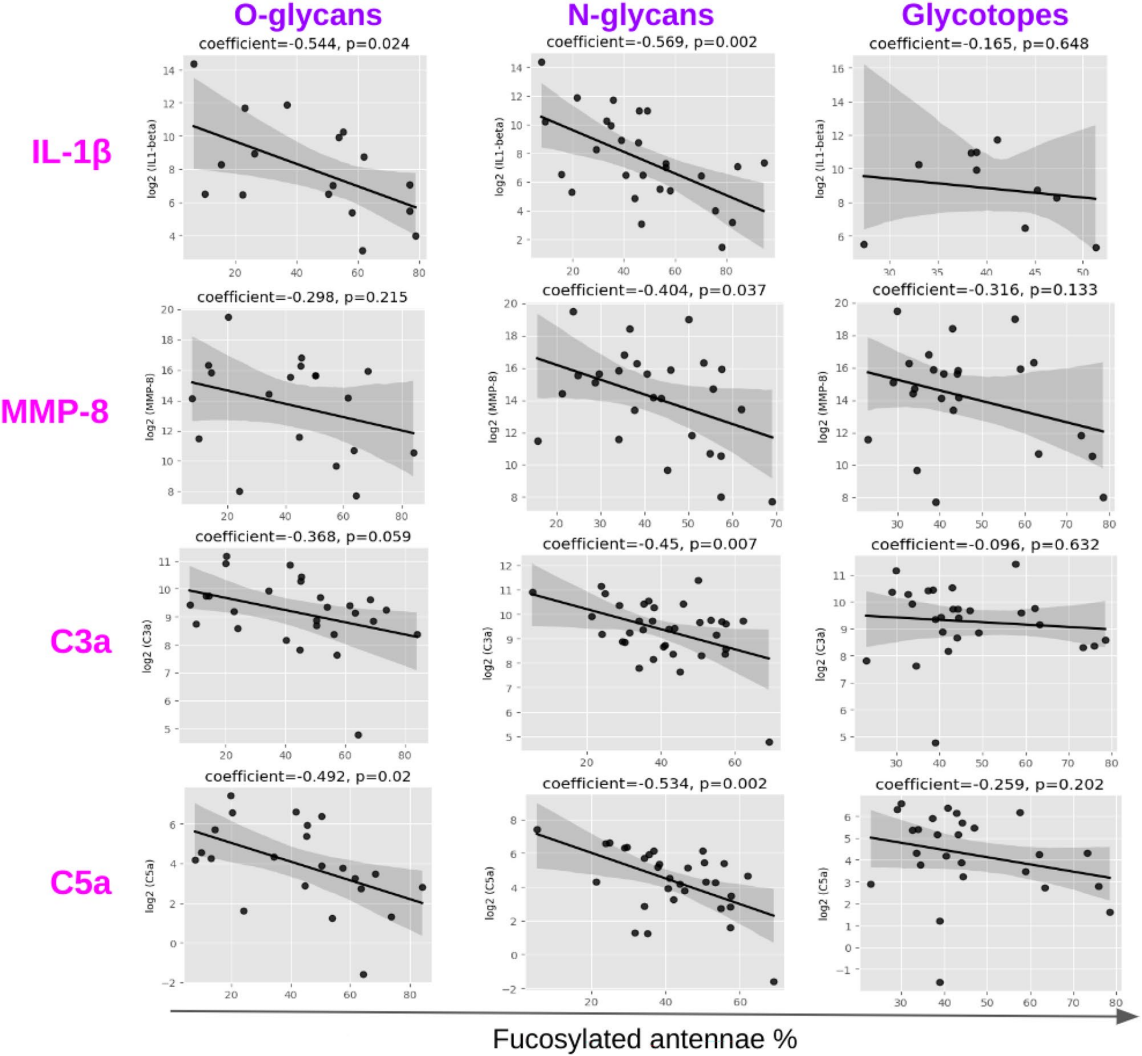
Correlation of glycans with fucosylated antennae to IL-1β, MMP-8, C3a and C5a. Fucosylated antennae % was calculated as the intensities of glycans or glycotopes with fucosylated antennae relative to the total complex glycan intensities in the spectra.

Glycans with fucosylated antennae, which are the source of Lewis and SLewis antigens, were negatively correlated with pro-inflammatory factors involved in neutrophil migration and activation, such as IL-1β, MMP-8, C3a and C5a in the cervicovaginal niche (Fig. 4). The correlation was stronger for N- and O-glycans than glycotopes. Similar trends were observed for other common pro-inflammatory cytokines, MMPs and complement proteins (Supplementary Figs S6–S8). By contrast, N-glycans, O-glycans and glycotopes with only sialylated antennae showed an opposite trend and were mainly positively correlated to these factors (Supplementary Figs S9–S11).

### Correlation of paucimannose glycans, ABO blood groups and LacdiNAc glycans with inflammatory mediators

**Fig. 5:**
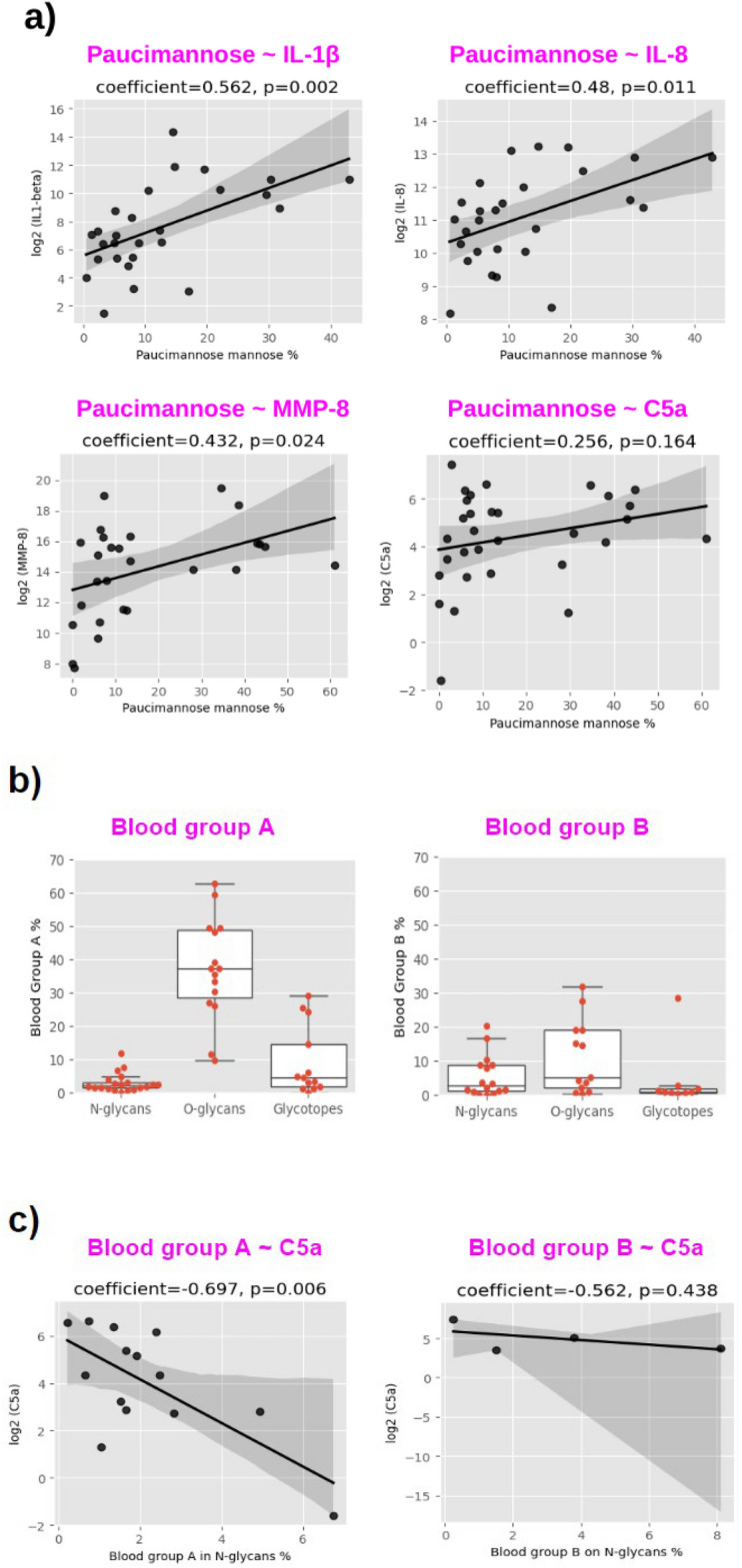
** a** Correlation of paucimannose glycans to IL-1β, IL-8, MMP-8 and C5a; **b** expression of blood group antigens; **c** correlation of blood group A and blood group B antigens to C5a. Paucimannose glycans % was calculated as the summed intensity of paucimannose glycans relative to the total intensity of paucimannose and high mannose glycans. Blood group A % in O-glycans is calculated as the summed intensity of blood group A on O-glycans in the total intensity of O-glycans in the spectrum. Blood group A in N-glycans % is calculated as the summed intensity of blood group A N-glycans in the total intensity of complex N-glycans in the spectrum. The same method was used to calculate Blood group B %.

Paucimannose glycans, which are known to be abundant in neutrophil granules^26–28^, showed a positive correlation to pro-inflammation, exemplified by IL-1β, IL-8, MMP-8 and C5a (Fig. 5a) and other pro-inflammatory factors (Supplementary Fig. S12). Blood group A and blood group B were identified on O-glycans, N-glycans and glycotopes (Fig. 5b). Patients within the same blood group type displayed variable levels of blood group antigen expression. For example, patients within blood group A type had blood group A antigen levels ranging from 10 to 60% in O-glycans. In addition, blood group antigens were found to be differentially expressed among N-glycans, O-glycans and glycotopes, with O-glycans having the highest expression. Further analysis implied that blood group A and blood group B could possibly have different correlation patterns to immune factors. For example, blood group A had a stronger correlation to C5a than blood group B on N-glycans (Fig. 5c). Analysis of other available data points showed that blood group A had negative correlation to MMPs and complement proteins but the correlations to cytokines was not strong (Supplementary Fig. S13). However, blood group B did not show clear correlation with complement proteins but showed a trend of negative correlation with cytokines (Supplementary Fig. S14). The LacdiNAc epitope was detected on N-glycans and confirmed by MS/MS analysis (see Supplementary Fig. S15). This structure was negatively correlated to MMPs, complement proteins, and inflammatory cytokines, with the exception of anti-inflammatory cytokine IL-4 (Supplementary Fig. S15).

### Glycosylation status and microbiota composition

**Fig. 6:**
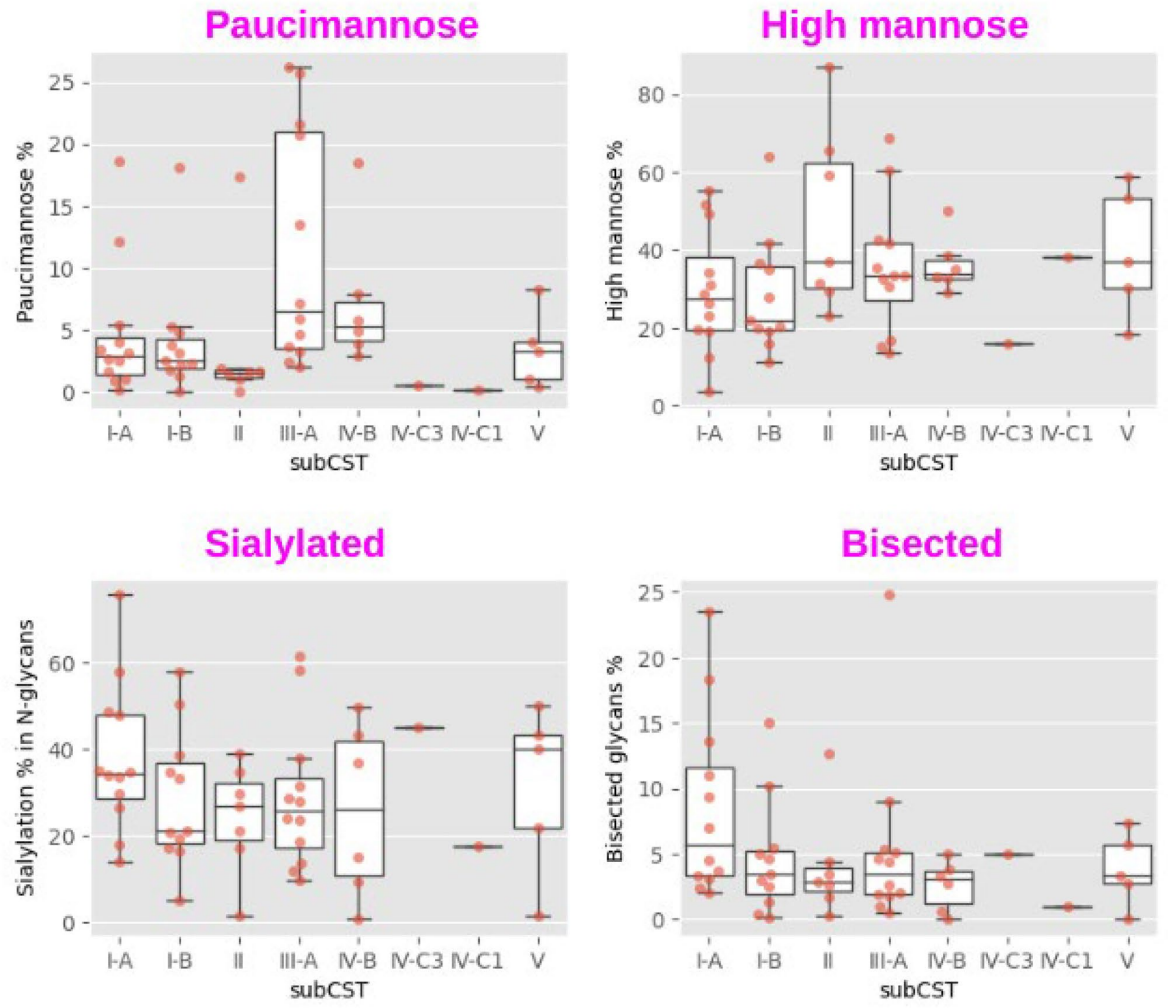
Relationship between CST status and paucimannose glycans, high mannose glycans, sialylated N-glycans and bisected glycans. Sialylation % was calculated as sialylated glycans in all N-glycans.

Glycosylation features that showed the most notable relationship with microbial composition are outlined below. Higher levels of paucimannose glycans were observed in microbiota communities CST III-A, dominated by *L. iners*, and CST IV-B, which contains a high to moderate relative abundance of *G. vaginalis* and *A. vaginae* (Fig. 6). Lower levels of high mannose glycans were observed in *L. crispatus* dominated samples (Fig. 6). Communities characterised by mostly *L. crispatus* (CST I-A) colonisation had the highest levels of sialylated and bisected glycans.

### Longitudinal glycomic profiling of CVF during pregnancy

**Fig. 7:**
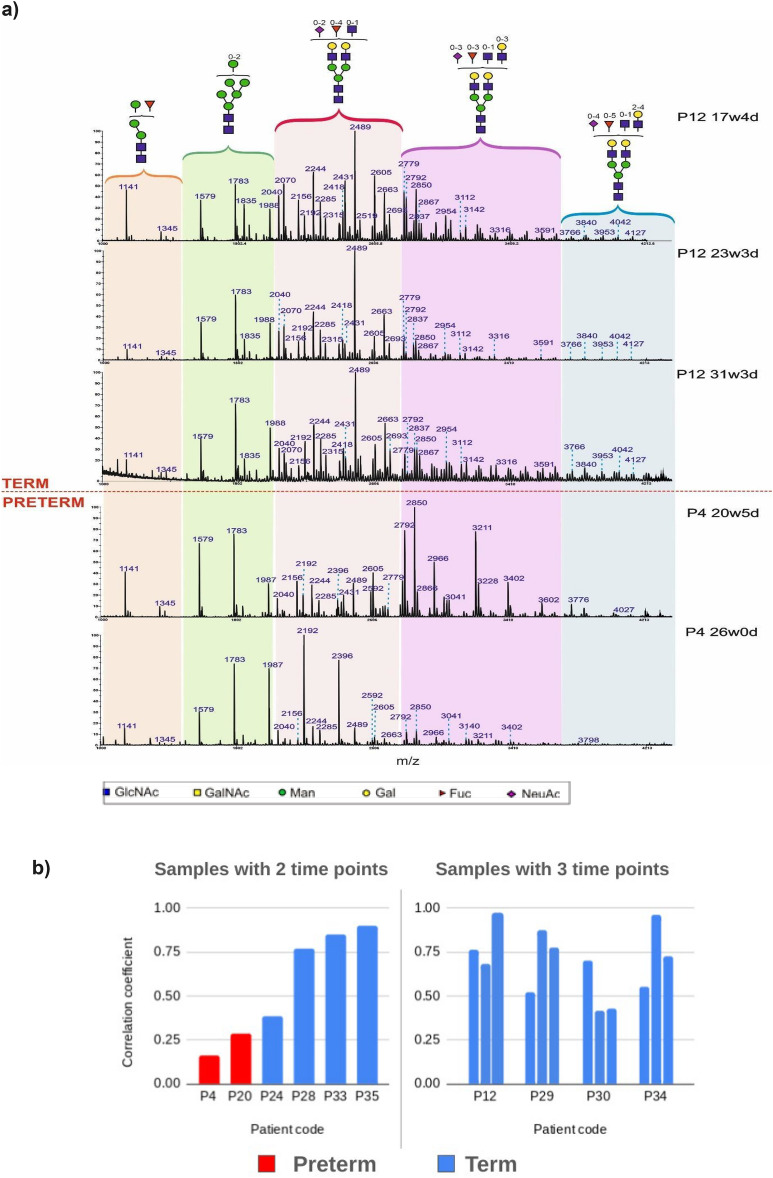
Preterm samples showed unstable N-glycosylation changes during pregnancy. **a** change of N-glycomic spectrum patterns during pregnancy of the term donor P12, a blood group O donor with CSTI-B who delivered at term at 37w6d, and the preterm donor P4, a blood group B donor with CST I-A who delivered preterm at 27w3d. Donor P12 had 3 samplings and donor P4 had 2 samplings during pregnancy. **b** Pearson coefficient values of donors with longitudinal samplings with mass range from m/z 1500 to 3500. A donor with 2 sampling time points has one coefficient value, while a donor with 3 sampling time points can have three coefficient values.

**Fig. 8:**
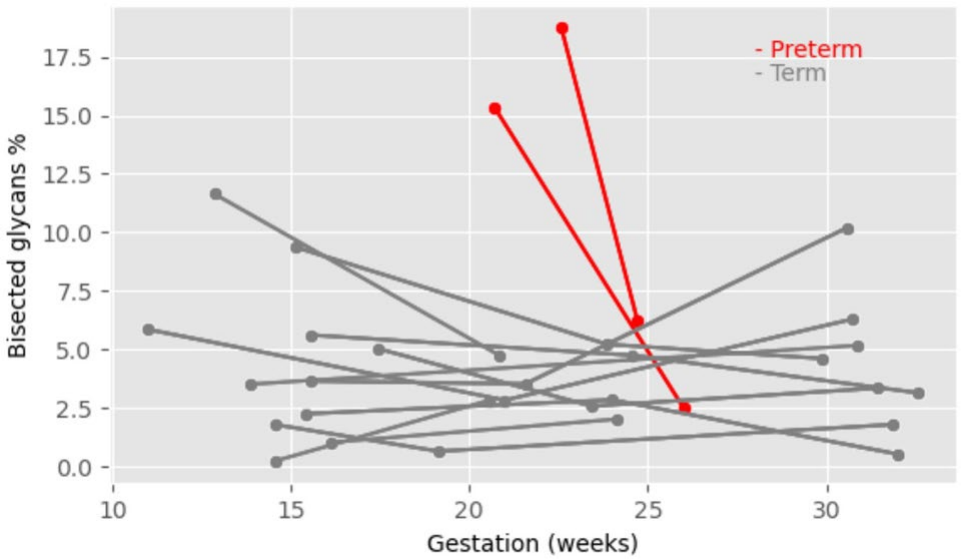
Change of bisected glycans in all longitudinal donors with samplings between 13 and 33 weeks. The preterm pregnancies showed a sharp decrease in bisected glycans. Relative intensity % was calculated as the summed intensities of bisected glycans in the total intensities of glycans at m/z range 1000–3500 of each spectrum.

**Fig. 9:**
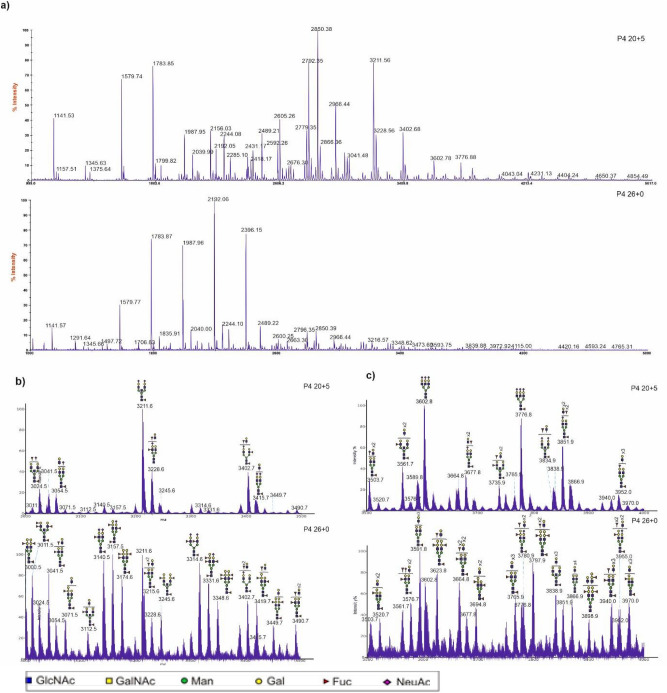
Longitudinal N-glycan spectra of sample P4 at 20w5d (upper panel) and 26w0d (lower panel), full spectra (m/z 1000–5000) are reported in panel **a**, while zoomed in sections are reported in panels **b,** m/z 3000–3500, and **c),** m/z 3500–4000. Cartoon structures of main peaks are depicted in panels **b** and **c**, Assignments are based on composition, tandem MS and knowledge of biosynthetic pathways. All molecular ions are [M + Na]^+^

CVF was sampled longitudinally (2 to 3 time points) throughout the pregnancies of 13 women, two of whom delivered extremely preterm. Analysis of N-glycan data indicated high stability of profiles for most term patients, but for both preterm patients this was not the case. For example, the CVF N-glycan profile for donor P12 remained highly stable across three sampling timepoints spanning the second and third trimesters of pregnancy (Fig. 7a). In contrast, donor P4, who delivered preterm, exhibited a major shift in the N-glycan profile between 20 and 26 weeks of pregnancy (Fig. 7a). Pearson correlation analysis indicated that women subsequently delivering preterm had very low coefficient values, indicative of large changes in their glycan profiles during pregnancy, whereas 8 of the 11 women delivering at term had high coefficient values (Fig. 7b, Supplementary Fig. S16). We also observed 3 term donors, which showed different coefficient values (Supplementary Fig. S17). Preterm donors also showed very high levels of bisected glycans at mid-trimester. Remarkably, the levels dropped dramatically shortly before the preterm birth (Fig. 8). In contrast, for women who went on to deliver at term, the values remained more stable. Interestingly, for preterm pregnancy P4, there was also a substantial increase in glycans carrying SLewis antigens, glycans carrying blood group B antigens, and glycans carrying both SLewis antigens and blood group B antigens just prior to preterm birth (Fig. 9, Supplementary Fig. S18).

## Discussion

This study reports the detailed characterisation of a broad range of glycan structures (N-glycans, O-glycans and their glycotopes) from CVF and their relationship with pregnancy status, microbiota composition and inflammatory and immune phenotypes. The work expands extensively on our previous description of N-glycosylation of CVF sampled during pregnancy, which was limited to 10 patients^25^. Validating our earlier findings, we observe a strong positive correlation between CVF sialylation and microbiota composition, and a negative correlation between high levels of fucosylation in CVF glycans and inflammatory cytokine concentrations (Supplementary Fig. S19–S26). The integration of O-glycan, N-glycan and glycotope datasets expands our characterisation of the CVF glycome and illuminates its complexity, characterised by diverse structures modified by different numbers of fucose and sialic acid, generating important antigens such as Lewis antigens, SLewis antigens and ABO blood groups. It should also be noted that the complexity of the CVF glycome does provide challenges for structural characterisation and that complementary analytical approaches, particularly those that also incorporate chromatography separation and ion mobility mass spectrometry could be of value for isomer separation and characterisation^29,30^. Functional correlation analysis of overall levels of glycan antenna sialylation and fucosylation also showed similar biological trends for N-glycans, O-glycans and glycotopes (Fig. 4). However, we did observe that CVF O-glycans had higher levels of blood group A and blood group B antigens than N-glycans and glycotopes (Fig. 5b). In addition, blood group A and blood group B appear to be correlated to immune factors in different ways (Fig. 5c).

Rich repertoires of high molecular weight fucosylated and sialylated glycans were found in all the pregnant CVF N-glycomes. In contrast, smaller N-glycans, notably paucimannose, high mannose and bisected biantennary complex-type, were found to be highly variable in their abundance amongst samples from pregnant donors (Fig. 1). The dominance of a particular family of low molecular weight glycans is most likely due to changes in the levels of specific glycoproteins in the CVF. CVF glycans can be derived from multiple sources, including epithelial cells in both vaginal and cervical regions, secreted mucins, antibodies, immune cells and liver derived glycoproteins abundant in amniotic fluid and serum^31^. Therefore, changes of individual protein levels could contribute specific glycan structures to the CVF glycome.

We found that a substantial number of pregnant CVF N-glycomes are dominated in the low mass region by minimally-processed high mannose glycans (Man_9_GlcNAc_2_ and Man_8_GlcNAc_2_; Fig. 1b, Supplementary Fig S27). These findings are consistent with our earlier results for non-pregnant CVF^25^. An abundant expression of high mannose N-glycans, and in particular the limited processing of Man_9_GlcNAc_2_ and Man_8_GlcNAc_2_ in the N-glycan biosynthetic pathway, is widely considered to be a feature of human cancer glycosylation^32–35^. This “abnormal” cancer high mannose glycosylation pattern is currently being targeted in the development of new cancer treatments by linking high mannose specific lectins to peptide toxin^36^, human IgG1 Fc^37^ or by generating lectin CAR-T cells^38^. A similar approach is being pursued for developing novel antiviral immunotherapies^39^, because again it is known that pathogenic human viruses such as SARS-CoV-2^40^ and Zika^41^ and HIV^42^ also express glycoproteins carrying high levels of Man-9 and Man-8 N-glycans. Our findings that high mannose glycosylation is a common characteristic of the CVF glycome stresses the need for careful assessment of potential off-target effects of anti-cancer or anti-viral therapeutics targeting such glycomic features.

Liver derived glycoproteins are considered to be major contributors to sialylated non-fucosylated N-glycans in the CVF^43^. There are two likely sources of these glycoproteins in the CVF: serum^44^ and amniotic fluid^45^. Therefore, high levels of sialylated non-fucosylated glycans could indicate leaking of serum proteins and/or amniotic fluid proteins to the cervicovaginal niche. Quantitative analysis showed sialylated non-fucosylated N-glycans were positively correlated to MMP levels, suggesting they are inflammation related, which was consistent with the correlation analysis to complement factors and cytokines (Supplementary Fig. S9–S11). MMPs are zinc-dependent proteases that degrade various proteins in the extracellular matrix and play important roles in vascular remodelling^46^. In the cervicovaginal region, however, MMPs can be a risk factor of pregnancy complications. Activation of extracellular matrix metalloproteinase causes the degradation of mucin, the disruption of the immune barrier to pathogens, eventually resulting in upper genital tract infections, inflammation and pregnancy complications^47,48^.

N- and O-glycans with fucosylated antennae were found to be negatively linked to inflammation (Fig. 4, Supplementary Fig. S6–S8). Fucosylated glycans are important immune regulators, which are involved in immune cell development, leukocyte adhesion and antigen presentation^49^. They are known to regulate host-microbiome interactions^50^, and studies on intestinal epithelial cells have found that they support the maintenance of symbiotic microorganisms and resistance to pathogens such as *Citrobacter rodentium*, *Salmonella typhimurium*, and *Enterococcus faecalis*. It is possible that the fucosylated glycan structures play similar roles in regulating the immune system and the microbiome in the CVF.

While ABO blood groups are important antigens on red blood cells, they are also expressed by epithelial cells, suggesting multiple biological roles for these antigens. Studies on human intestinal epithelium have found that ABO blood group antigens are genetically determined host factors that can be recognized by bacterial adhesins and modulate the composition of microbiota^51,52^. Studies on the human genital tract have discovered that blood group B can be a risk factor in the development of neonatal group B streptococcal disease^53^, and loss of blood group A can be linked to the progression of squamous intraepithelial lesions^54^. In the present study, we used a glycomic strategy to directly investigate ABO blood group antigens and identified these antigens on N-glycans, O-glycans and extended antennae (Figs. 2, 3, supplementary Fig. S18). More importantly, we discovered that patients with the same blood group genotype can have very different expressions of blood group antigens. Moreover, blood group antigen levels are different among N-glycans, O-glycans and glycotopes, with O-glycans having the highest blood group antigen levels (Fig. 5b). Affinity enrichment using anti-blood group antibodies followed by quantitative proteomics could be used in future studies to identify the key glycoproteins that carry the blood group antigens.

The activity of the host immune system must be delicately balanced during pregnancy. A loss of balance can cause pregnancy complications. Our longitudinal glycomic data showed stable glycosylation during pregnancy in most term birth donors (Fig. 7), which could be an indicator for good maintenance of immune balance. By contrast, in the preterm group, there was a big glycosylation change within a short time, which could indicate a disruption of immune homeostasis (Fig. 7).

Bisected glycans have a distinctive structural feature: a non-extended GlcNAc β1-4 linked to the core mannose, which has a big impact on the conformation of the glycan structure^55^. Bisected glycans have been found to be involved in immune tolerance by suppressing NK cells^56,57^. NK cells are the major type of immune cells in the maternal-foetal interface^58^, where bisected glycans are also highly expressed, and are believed to protect the foetus from immune rejection^59^. However, the major immune cell population in the cervicovaginal region are neutrophils^60–62^, not NK cells, indicating additional functionality. We observed large variations in the levels of bisected glycans, which dominated the spectra of some CVF samples, but were not detected in some other samples, suggesting they could be derived from specific glycoprotein carriers. It is known that bisected glycans are not abundant in neutrophils^63^, liver derived proteins^64^ or antibodies^65,66^. Therefore, these constituents of CVF are unlikely to be the source of the abundant bisected glycans that are observed in some samples. On the other hand, mucins are amongst the most abundant glycoproteins in the CVF. A large-scale identification of mucins in endocervical mucus previously identified three gel-forming mucins (MUC5B, MUC5AC, and MUC6) and two transmembrane mucins (MUC16 and MUC1)^67^. The gel forming mucins and MUC1 are predominantly glycosylated with O-glycans and have only a limited number of N-glycosylation sites. Thus, they are not good candidates as carriers of the abundant bisected glycans. In contrast, MUC16, which is the largest mucin with a ~ 22,152 amino acid sequence, has as many as 250 potential N-glycosylation sites^68^. It has been found to be highly expressed in the cervix and its extracellular glycosylated domains can be shed from the epithelial cells by proteolysis^69^. In addition, the proteolytically cleaved soluble form of ovarian MUC16, which is also known as CA125 and is used for cancer screening, is characterised by high levels of bisected glycans^70^. These observations suggest that a potential source of the bisected glycans we identified in the CVF is cervical MUC16.

Correlation analysis showed that bisected glycans had a higher relative abundance in CST I-A than other CST subtypes (Fig. 6). In addition, expression of bisected glycans was generally stable in women experiencing term births whereas a sharp decrease in their levels was observed before delivery in women experiencing preterm birth (Fig. 8). These results suggest that MUC16 levels could be correlated to the cervicovaginal microbiome and preterm birth. A knockdown of MUC16 in an epithelial cell line was shown to decrease all barrier functions, exemplified by increased dye penetrance and bacterial invasion, decreased transepithelial resistance, disruption of tight junctions, and greater apical surface cell area. By contrast, knockdown of MUC1 did not cause an obvious barrier loss^71^.

In summary, this study demonstrates important relationships between CVF glycans and microbiota-host immune interactions, which are risk modifiers of pregnancy outcome. Detecting changes in CVF glycosylation during pregnancy could provide a new, non-invasive method for identifying women who are at risk of preterm birth and therefore implementation of personalised interventions. Additionally, understanding the role of glycans during pregnancy will help uncover the causes of preterm birth in women without a known aetiology. This knowledge can lead to the development of new interventions and glycan-mimicry based therapies that have the potential to reduce levels of preterm birth and improve the health outcomes of mothers and their babies.

## Materials and methods

### Patient recruitment and sampling

The study was conducted with approval of the NHS National Research Ethics Service (NRES) Committees London—Stanmore (REC 14/LO/0328), and in accordance with relevant guidelines, regulations, and the Declaration of Helsinki. All pregnant and non-pregnant women provided written informed consent. Recruitment and sampling were performed at Queen Charlotte’s and Chelsea and Westminster Hospitals, Imperial College Healthcare NHS Trust, London, UK. Non pregnant women were eligible if they were of reproductive age and aged 18 or over. Pregnant women at risk of preterm birth were eligible. Risk factors included having a short (cervical length of ≤ 25 mm) or open cervix, a previous preterm delivery, mid trimester loss, history of preterm premature rupture of the membranes, previous fully dilated caesarean section, or previous excisional cervical treatment. Exclusion criteria included women under 18 years of age, those who had sexual intercourse within 72 h of sampling, vaginal bleeding in the preceding week, HIV or Hepatitis C positive status. Detailed maternal clinical metadata and birth outcome data was collected for all pregnant participants (Supplementary Table S1). CVF was sampled using the BBL™ CultureSwab™ MAXV liquid Amies swabs (Becton, Dickinson and Company, Oxford UK) for assessing microbial composition. Supernatant from the culture swab was also used for immune profiling assays. CVF was then collected using a menstrual cup (SoftdiscTM, The Flex Company, USA) by placing it against the cervix for 20 min. After removal, material from both sides of the cup was retrieved by repeated pipetting of phosphate buffer saline (PBS) over each side leading to resuspension of material in a 1:5 weight: volume ratio within 30 min of collection. The suspension was distributed into separate aliquots to prevent unnecessary free thaw cycles. Samples were stored at − 80 °C until analysis.

### Glycan profiling of CVF samples

Methanol, acetonitrile, ammonia, chloroform, DMSO, propan-1-ol, sodium hydroxide and acetic acid were from Romil (Cambridge, UK). Iodoacetic acid, sodium chloride, iodomethane, ammonium bicarbonate, EDTA, trypsin, Tris, potassium hydroxide, potassium borohydride, sodium acetate, sodium borodeuteride and Dowex 1-X8 beads were from Merck (Poole, UK). PNGase F (cloned from *Flavobacterium meningosepticum* and expressed by *E. coli*), CHAPS and DTT were from Roche Applied Science (East Sussex, UK). 8 M guanidine hydrochloride (GuHCl) and Slide-A-Lyzer™ G2 Dialysis Cassettes, 3.5 K MWCO were from Thermo Scientific (Loughborough, UK). Endo-Beta-Galactosidase (*F. keratolyticus*) was from R&D Systems (Abingdon, UK).

The workflow for glycomic sample processing is illustrated in Supplementary Fig. S28, following the protocols detailed previously^72,73^. Briefly, CVF samples were sonicated in 25 mM Tris, 150 mM NaCl, 5 mM EDTA, and 1% CHAPS, pH 7.4, dialysed in dialysis cassettes, reduced by DTT, carboxymethylated by IAA, and digested by trypsin.

### N-Glycans

N-glycans were released by PNGase F, separated from O-glycopeptides by C18 Sep-Pak chromatography and permethylated following the NaOH procedure. The permethylated N-glycans were cleaned by C18 cartridges and freeze dried before mass spectrometry analysis.

### O-Glycans

Four hundred microliters of 0.1 M potassium hydroxide containing potassium borohydride (54 mg/ mL) was added to dried samples and incubated at 45 °C for 14–16 h. The reaction was terminated by adding a few drops of 5% (v/v) acetic acid followed by purification with Dowex 1-X8 desalting column. Excess borates in the samples were subsequently removed by co-evaporating with 10% (v/v) acetic acid in methanol under a stream of nitrogen at room temperature. The purified native O-glycans were then permethylated following the NaOH procedure.

### Glycotopes

Parallel to full-size N- and O-glycan analysis, glycotopes from extended antennae on N- and O-glycans were removed from glycopeptides by incubating with 8 ul (4 ug) endo-β-galactosidase (Supplementary Fig. S29) in 400 ul 50 mM sodium acetate buffer, pH 5.5–6, at 37 ˚C for 24 h. After digestion, 3 drops of acetic acid were added to the samples. After C18 Sep-Pak chromatography, the isolated, freeze-dried glycotopes were deuteroreduced by incubating with a 10 mg/ml solution of sodium borodeuteride in 2 M ammonia for 2 h at room temperature. The reaction was stopped by addition of 5 drops of acetic acid and the samples were dried under nitrogen. Excess borates were removed by co-evaporating with 10% (v/v) acetic acid in methanol under a stream of nitrogen at room temperature. The deuteroreduced glycotopes were then permethylated following the NaOH procedure and analysed by MALDI-TOF–MS and MS/MS analysis. It should be noted that in our previous publication^25^ glycotopes were analysed on an advanced Orbitrap Fusion Tribrid MS platform with nanoLC-MS^2^-product dependent-MS^3^ data acquisition workflow. This allowed isomeric glycotopes such as Lewis X/A to be differentiated. Such levels of differentiation are not possible with the MALDI-TOF–MS and MS/MS analysis.

### GC/MS linkage analysis

GC–MS linkage analysis of partially methylated alditol acetates was carried out on a Bruker SCION 456-GC Gas Chromatograph, fitted with a BR-5 ms fused capillary column (15 m × 0.25 mm internal diameter, Bruker), coupled to a Bruker SCION SQ Mass Spectrometer. Partially methylated alditol acetates were prepared from permethylated samples as described previously^71^. The permethylated glycans were hydrolyzed with 2 m trifluoroacetic acid for 2 h at 121 °C, reduced with 10 mg/ml sodium borodeuteride in 2 m aqueous ammonium hydroxide at room temperature, and acetylated with acetic anhydride at 100 °C for 1 h. The sample was dissolved in hexanes and injected onto the column at 60 °C. The column was maintained at this temperature for 1 min and then heated to 300 °C at a rate of 8 °C/min.

### MALDI-MS and MS/MS analysis

Glycan profiling was done on an AB Sciex 4800 MALDI-TOF/TOF mass spectrometer. The methylated glycans and glycotopes were dissolved in 10 ul methanol. 1ul of sample was mixed with 1 ul of 10 mg/ml DABP matrix in 75% ACN. The mixture was spotted on a MALDI plate for MALDI-TOF–MS and MS/MS analysis.

### Glycomic data analysis

The data were analysed using Data Explorer™ version 4.6 from AB Sciex, Glycoworkbench^74^ and MALDIquant^75^. The glycomic data were annotated based on monosaccharide composition derived from the molecular ion m/z value, knowledge of glycan biosynthetic pathways, the isotopic peak cluster patterns, the glycosylation patterns in the low and medium mass range, and MS/MS derived fragmentation. An R programme (https://github.com/gw110/Glycomics-of-cervicovaginal-fluid-from-women-at-risk-of-preterm-birth.git) was developed to quantify glycans in overlapped mono-isotopic peak clusters of multiple glycans. A multinomial distribution model was first trained using standard individual permethylated glycans and the molecular formula of these glycans. The optimised model was then used for deisotoping of overlapped mono-isotopic peak clusters by matching the predicted monoisotopic peak patterns of individual glycans to the overlapped peaks. An overdetermined linear system was constructed, and the intensities of individual glycans were calculated by solving the linear system with least squares. Glycan matches with R-squared values above 0.9 were selected for further analysis.

The correlation analysis of glycans and data visualisation were done using Python 3.10.9 on Linux Debian 11, and the script is available on (https://github.com/gw110/Glycomics-of-cervicovaginal-fluid-from-women-at-risk-of-preterm-birth.git). The correlation of glycan relative intensities with MMPs, Complements and cytokines were done using the linear regression model. Pearson product-moment correlation coefficient and p value were calculated to evaluate the strength of the correlation.

### Bacterial DNA extraction and metataxonomic profiling

Extraction of DNA from CVF samples and sequencing of 16S rRNA hyper variable regions was performed as previously described^76^. Briefly, V1-V2 hyper variable regions of bacterial 16S rRNA genes were amplified using a mixed forward primer set (28f.-YM) consisting of the following primers mixed at a 4:1:1:1 ratio; 28F-Borrellia GAGTTTGATCCTGGCTTAG; 28F-Chlorflex GAATTTGATCTTGGTTCAG; 28F-Bifido GGGTTCGATTCTGGCTCAG; 28F GAGTTTGATCNTGGCTCAG. The reverse primer consisted of; 388R TGCTGCCTCCCGTAGGAGT^77^. Amplified products were then pooled equimolar and each pool was size selected in two rounds using Agencourt AMPure XP (BeckmanCoulter, Indianapolis, Indiana) in a 0.7 ratio for both rounds. These were then quantified using the Quibit 2.0 Fluorometer (Life Technologies) and loaded on an Illumina MiSeq (Illumina, Inc. San Diego, California) 2 × 300 flow cell at 10 pM. All sequencing was performed at Research and Testing Laboratory (Lubbock, TX, USA).

Trimming of primer sequences was performed using Cutadapt^78^ and QC performed using FastQC^79^. Resulting amplicon sequence variant (ASV) counts were calculated for each sample using the Qiime2 pipeline^80^. DADA2 was used for denoising^81^ and taxonomic classification of sequences to species level was performed using the STIRRUPS reference database^82^. Samples were classified into vaginal community state types (CSTs) using the VAginaL community state typE Nearest CentroId classifier (VALENCIA)^83^. Using this standardised approach, CST I communities are characterised by *Lactobacillus crispatus* dominance and can be further subdivided into subtypes CST I-A (almost complete dominance by *L. crispatus*) and CST I-B (less *L. crispatus*, but still the majority). CST IV communities have a low relative abundance of Lactobacillus spp. and include subtypes CST IV-A, CST IV-B, and CST IV-C, depending on the relative abundances of *G. vaginalis*, *A. vaginae*, and BVAB1, respectively.

### Immune profiling of CVF samples

The stored supernatant of CVF from the menstrual cup was thawed on ice and used for Luminex® immunoassays to quantify cytokines levels of IL-2, IL-4, IL-18 using the Human Premixed Multi-Analyte Kit (R&D Systems). Further immune profiling was performed using supernatant extracted from the BBL™ CultureSwab™ to quantify the other immune mediators by multiplexed bead-based immunoassays. The concentrations of cytokines (IL-1β, IL-6) were measured on a multiplex plate Human Premixed Multi-Analyte Kit (R&D Systems), following manufacturer’s instructions. IL-8 was measured on a single-plex Human Premixed Analyte Kit (R&D Systems), following a tenfold dilution using Calibrator Diluent RD6-52. The concentrations of MMPs (MMP-2, MMP-8, MMP-9) were measured on a Human Premixed Multi Analyte Kit (R&D Systems) following a fivefold dilution using Calibrator Diluent RD6-52. Complement proteins were detected using the Human Complement Magnetic Bead Panel 1 (C2, C4b, C5, C5a, Factor D, Factor I, MBL) and Human Complement Magnetic Bead Panel 2 (C3, C3b, C4, Factor H) (Milliplex® Meck/Millipore). C3a was measured using the C3a Human ProcartaPlex™ Simplex Kit in conjunction with ProcartaPlex Human Basic Kit (both Invitrogen™). All standards and samples were run in duplicate.

## Supplementary Information

Supplementary Information.

## Data Availability

The glycan libraries, glycan isotopic peak matches, glycan identifications, R script for deisotoping and Python script for data visualisation are available on Github: https://github.com/gw110/Glycomics-of-cervicovaginal-fluid-from-women-at-risk-of-preterm-birth.git The datasets used and/or analysed during the current study available from the corresponding author on reasonable request.
