# Supplementary material for: Glycomics of cervicovaginal fluid from women at risk of preterm birth reveals immuno-regulatory epitopes that are hallmarks of cancer and viral glycosylation
Source: Sci Rep. 2024 Sep 6;14:20813. doi: 10.1038/s41598-024-71950-x (PMC11379862; doi:10.1038/s41598-024-71950-x)
Supplement: Supplementary file 1 — Supplementary Information. [file 41598_2024_71950_MOESM1_ESM.docx]

# Supplementary data

Supplementary Table S1. Summary of demographics of CVF samples

| All | **Preterm** | **Term** | **NP** | **p-value** |
| --- | --- | --- | --- | --- |
| **N (%)** | 15 (37.5%) | 21 (52.5%) | 4 (10%) |  |
| **Age (mean, IQR)** | 31 (26-34) | 34 (29-37) | Not recorded | 0.15 |
| **Ethnicity** |  |  |  |  |
| White | 5 (33.33%) | 13 (61.91%) | 2 (50%) | 0.31 |
| Black | 7 (46.67%) | 3 (14.29%) | 1 (25%) | 0.09 |
| Other | 3 (20%) | 4 (19.05%) | 1 (25%) |  |
| **BMI (mean, IQR)** | 24.21 (20-27) | 28.36 (22-35) | Not recorded | 0.06 |
| **ABO blood group** |  |  |  | 0.07 |
| A | 2 (13.33%) | 8 (38.1%) | 3 (75%) |  |
| B | 8 (53.33%) | 4 (19.05%) | 0 (0%) |  |
| O | 5 (33.33%) | 9 (42.86%) | 1 (25%) |  |
| **Gestational Age Delivery, Mean(IQR)** | 28+3 (22+4-34+4) | 39+1 (38+2-39+5) | n/a | <0.0001 |
| **Risk factor for PTB** |  |  |  |  |
| Previous MTL/ PTB | 5 | 12 | n/a |  |
| Previous cervical excisional treatment | 1 | 4 | n/a |  |
| Incidental finding of a short cervix | 8 | 0 | n/a |  |
| Multiple risk factor | 0 | 1 | n/a |  |
| Other* | 1 | 4 | n/a |  |
| * Fully dilated caesarean section, Previous cerclage, Uterine anomaly | | | | |

Supplementary Table S2. Summary of glycomic datasets obtained

| Patient code - sample collection | N-glycans | O-glycans | Glycotopes |
| --- | --- | --- | --- |
| NP1-0plus0 | ✓ | ✕ | ✕ |
| NP2-0plus0 | ✓ | ✓ | ✕ |
| NP3-0plus0 | ✓ | ✕ | ✕ |
| NP4-0plus0 | ✓ | ✓ | ✕ |
| P1-15plus3 | ✓ | ✕ | ✓ |
| P10-20plus2 | ✓ | ✓ | ✕ |
| P11-20plus2 | ✓ | ✓ | ✓ |
| P12-17plus3 | ✓ | ✓ | ✓ |
| P12-23plus3 | ✓ | ✓ | ✓ |
| P12-31plus3 | ✓ | ✓ | ✓ |
| P13-22plus3 | ✓ | ✓ | ✕ |
| P14-18plus4 | ✓ | ✓ | ✕ |
| P15-22plus2 | ✓ | ✓ | ✕ |
| P16-19plus5 | ✓ | ✓ | ✕ |
| P17-24plus2 | ✓ | ✓ | ✓ |
| P18-14plus6 | ✓ | ✓ | ✓ |
| P19-20plus4 | ✓ | ✕ | ✓ |
| P2-22plus5 | ✓ | ✓ | ✕ |
| P20-22plus4 | ✓ | ✓ | ✓ |
| P20-24plus5 | ✓ | ✓ | ✓ |
| P21-20plus3 | ✓ | ✓ | ✓ |
| P22-21plus2 | ✓ | ✓ | ✓ |
| P23-10plus3 | ✓ | ✓ | ✕ |
| P24-13plus6 | ✓ | ✓ | ✓ |
| P24-30plus6 | ✓ | ✓ | ✓ |
| P25-16plus1 | ✓ | ✓ | ✓ |
| P26-13plus6 | ✓ | ✓ | ✓ |
| P27-31plus3 | ✓ | ✓ | ✓ |
| P28-12plus6 | ✓ | ✓ | ✓ |
| P28-20plus6 | ✓ | ✓ | ✓ |
| P29-15plus4 | ✓ | ✓ | ✓ |
| P29-21plus4 | ✓ | ✓ | ✓ |
| P29-30plus4 | ✓ | ✕ | ✓ |
| P3-20plus4 | ✓ | ✓ | ✕ |
| P30-15plus4 | ✓ | ✓ | ✓ |
| P30-24plus4 | ✓ | ✓ | ✓ |
| P30-32plus4 | ✓ | ✓ | ✓ |
| P31-15plus3 | ✓ | ✓ | ✓ |
| P31-24plus0 | ✓ | ✓ | ✓ |
| P31-32plus0 | ✓ | ✕ | ✓ |
| P32-11plus0 | ✓ | ✓ | ✓ |
| P32-21plus0 | ✓ | ✓ | ✓ |
| P32-30plus5 | ✓ | ✓ | ✓ |
| P33-16plus1 | ✓ | ✓ | ✓ |
| P33-24plus1 | ✓ | ✕ | ✓ |
| P34-14plus4 | ✓ | ✓ | ✓ |
| P34-19plus1 | ✓ | ✓ | ✓ |
| P34-31plus6 | ✓ | ✕ | ✓ |
| P35-14plus4 | ✓ | ✓ | ✓ |
| P35-20plus4 | ✓ | ✓ | ✓ |
| P36-15plus1 | ✓ | ✓ | ✓ |
| P36-23plus6 | ✓ | ✓ | ✓ |
| P36-29plus6 | ✓ | ✓ | ✓ |
| P4-20plus5 | ✓ | ✓ | ✓ |
| P4-26plus0 | ✓ | ✓ | ✕ |
| P5-23plus5 | ✓ | ✓ | ✓ |
| P6-21plus3 | ✓ | ✓ | ✕ |
| P7-22plus5 | ✓ | ✓ | ✓ |
| P8-22plus0 | ✓ | ✓ | ✕ |
| P9-25plus3 | ✓ | ✓ | ✓ |

✓: data available ✕: data not available

Supplementary Table S3. 10 most abundant partially methylated alditol acetates obtained from GC-MS analysis of the 50% acetonitrile fraction of PNGase F released N-glycans of sample P2.


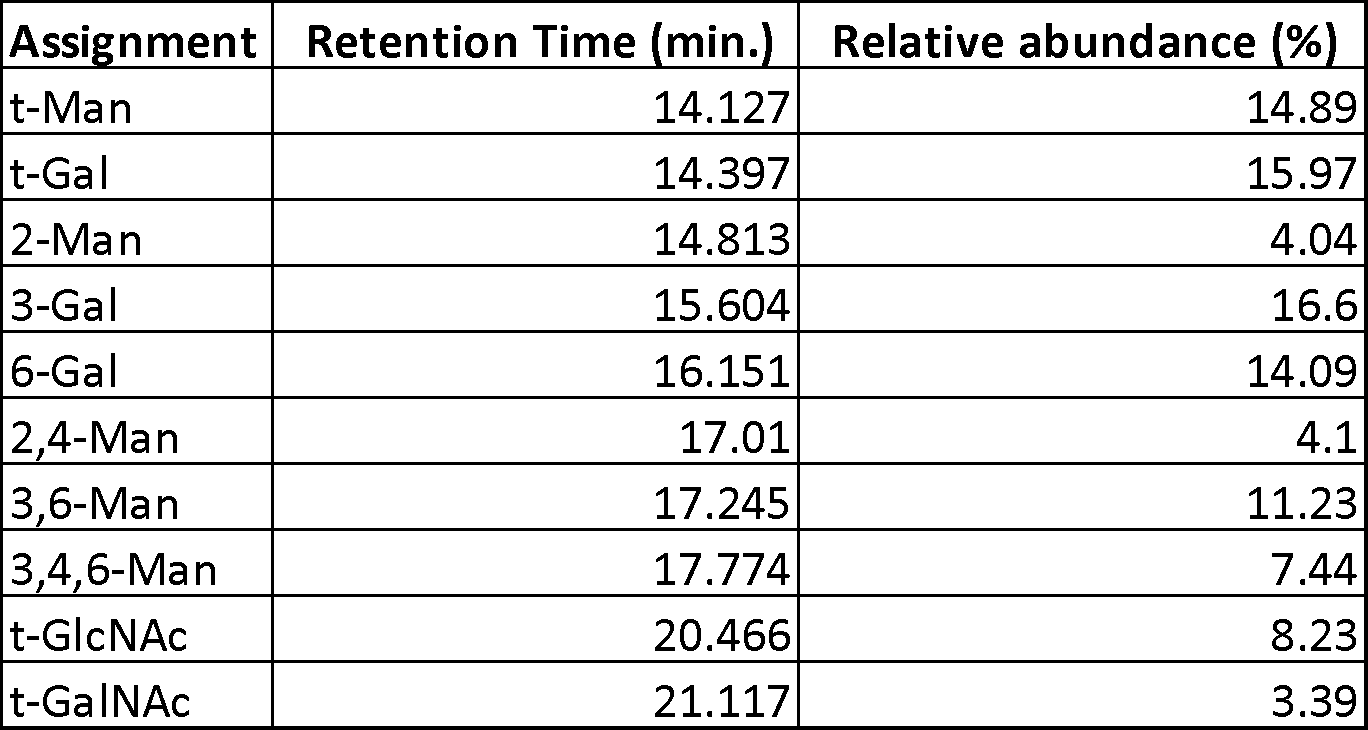


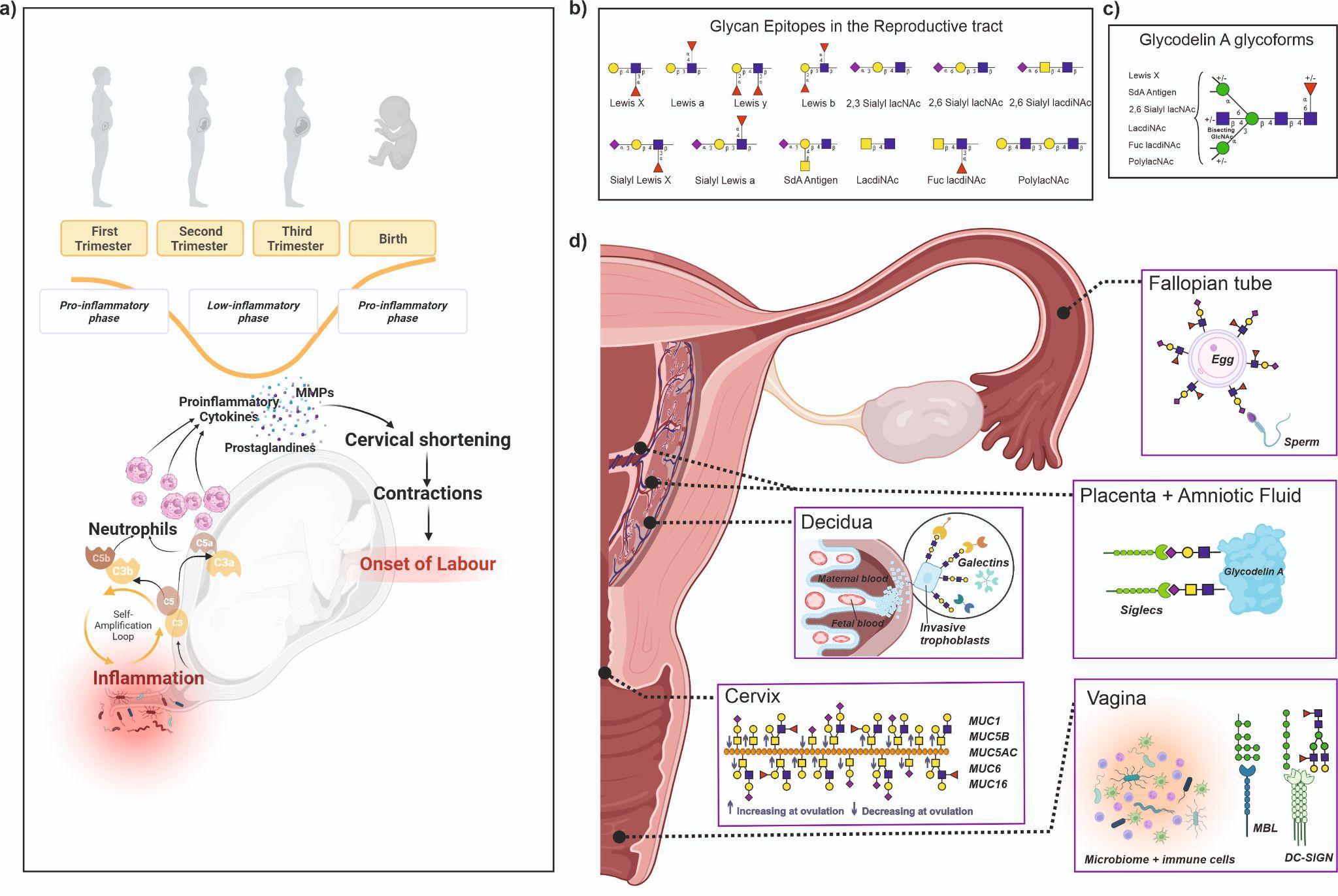


Supplementary Fig. S1. **a)** inflammation curve during pregnancy and onset of labour: transition from a pro-inflammatory state during the first trimester, to a low-inflammatory state during the second trimester, and to a pro-inflammatory state in late gestation. Inflammation in the lower reproductive tract activates a self-amplification loop, which leads to recruitment and activation of leukocytes, release of proinflammatory cytokines and to ultimately, the onset of labour; **b)** glycan epitopes found in the human female reproductive tract; **c)** Glycodelins are the best characterised immunomodulatory glycoproteins in the female reproductive tract. Glycodelin A (GdA) is expressed by endometrial and decidual cells and is found in both amniotic fluid and placenta. GdA displays many of the epitopes shown in a) on its N-linked glycans; **d)** representation of the main known glycan-lectin interactions along the human female reproductive tract, in the fallopian tubes, in the placenta and amniotic fluid, in the decidua and in the cervix and vagina. Figure created in **Biorender.**com.


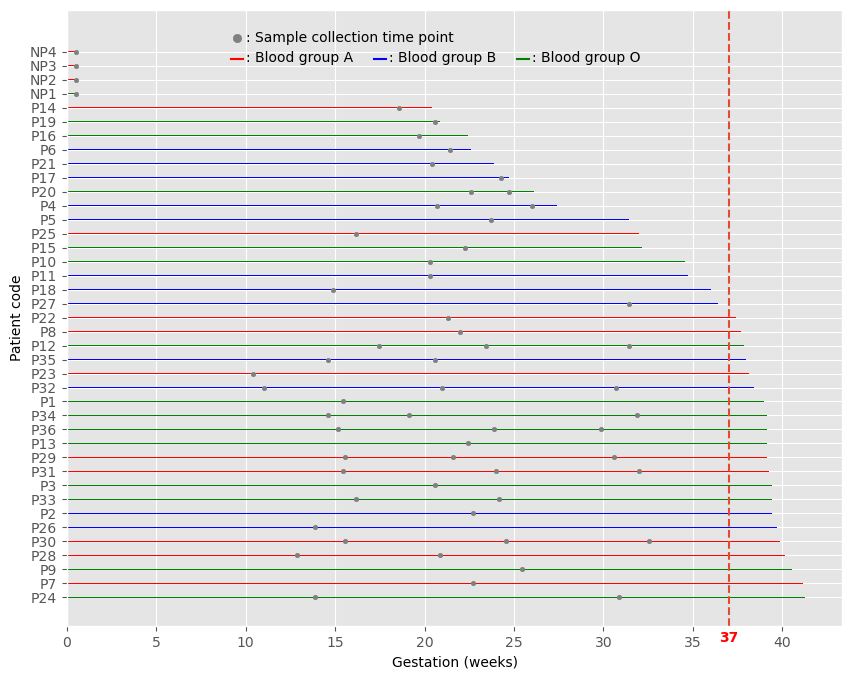


Supplementary Fig. S2. The sample cohort for glycomic analysis. The length of the horizontal lines shows the length of gestation (weeks). The dots on the lines show the sample collection time points. The colours of the lines show the ABO blood group. The patient code is shown on y axis.


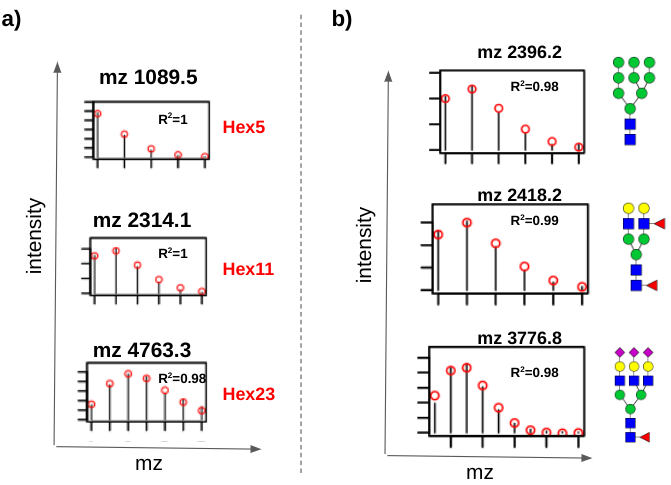


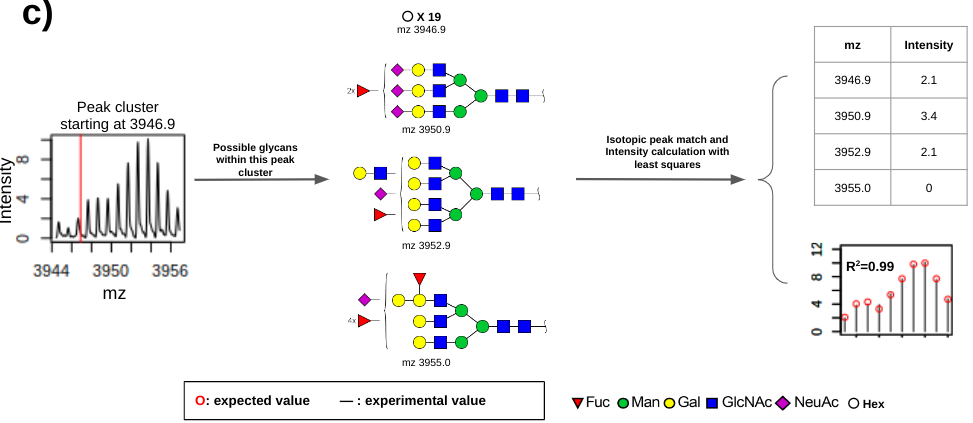


Supplementary Fig. S3. Prediction of mono-isotopic peak patterns of poly hexoses **a)** and N-glycans **b)** using an in-house R programme, both of which showed accurate prediction. Deisotoping an overlapped mono-isotopic peak cluster of 4 potential glycans from a blood group A CVF sample **c)**. The mono-isotopic peak pattern of each possible glycan was predicted and matched to the overlapped peak cluster with least squares, through which the intensity of each glycan was calculated. R squared is used to measure the quality of fit. All molecular ions are [M+Na]^+^.


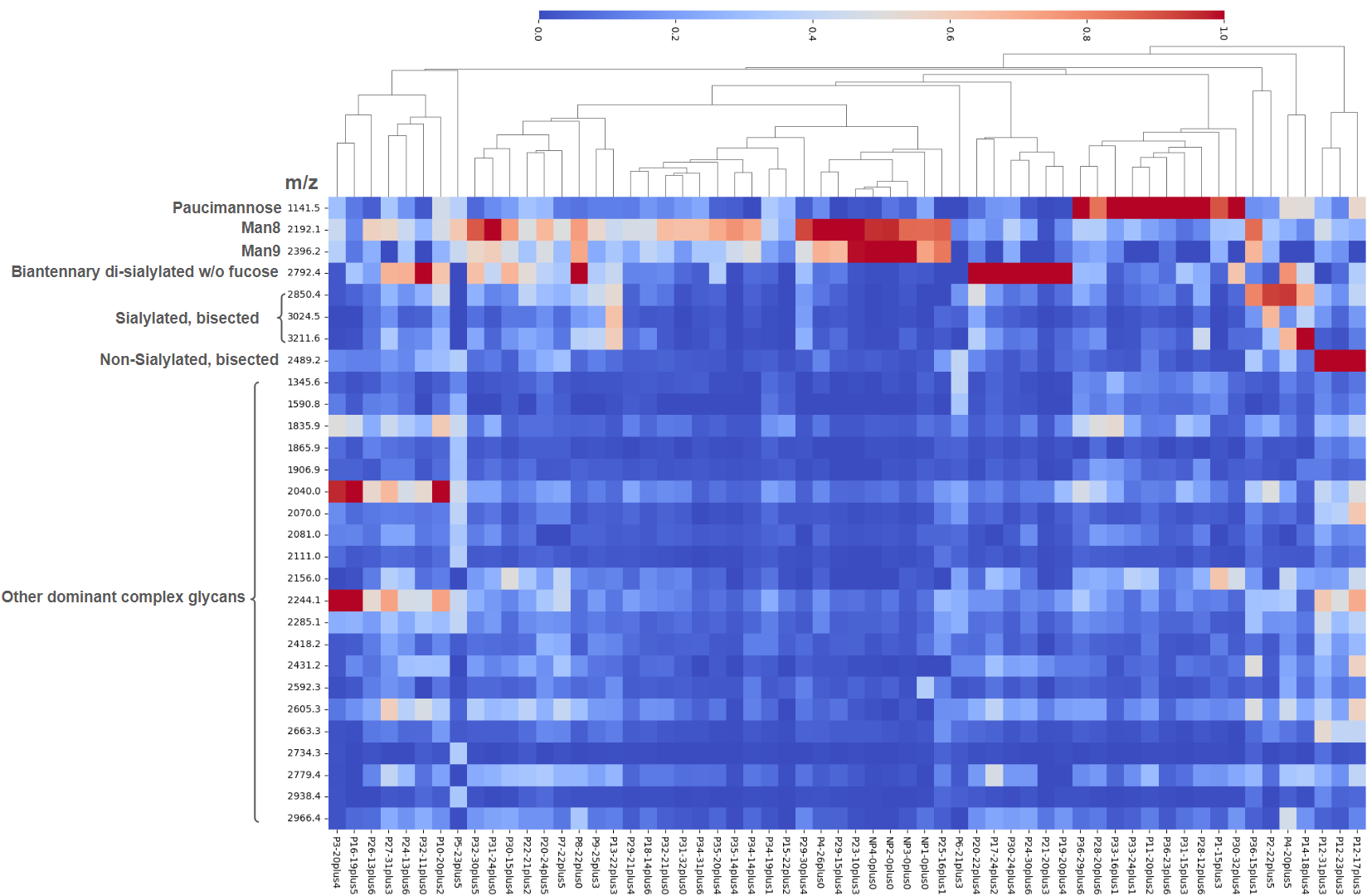


Supplementary Fig. S4. Hierarchical clustering of the CVF samples according to the relative intensities of paucimannose, Man8-9, bi-antennary di-sialylated glycan without fucose, bisected glycans and other dominant complex glycans. A few spectra with high relative intensities of truncated and non-sialylated bi-antennary glycans (m/z 1835.9, m/z 2040.0, m/z 2244.1) were observed, which might reflect IgG specific glycans or complex glycans truncated by vaginal microbes. Relative intensities were calculated as the ratio of an individual glycan signal to the highest signal in the spectra. Relative intensities below 30% in all samples were excluded.


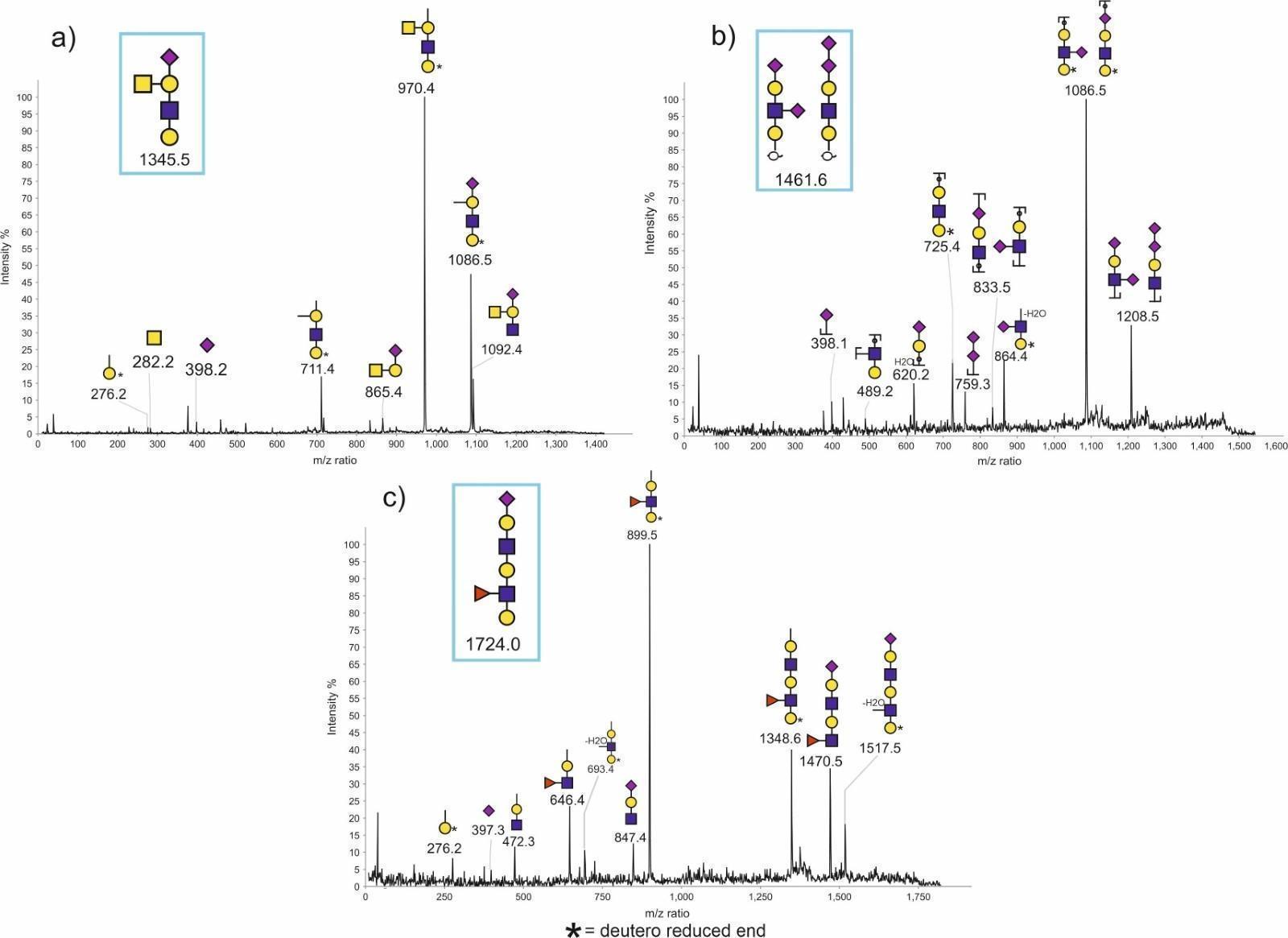


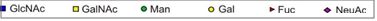


Supplementary Fig. S5. MS/MS spectra of released deutero-reduced and permethylated glycotopes. **a)** MS/MS spectrum of molecular ion at m/z 1345 from sample P36. The fragments at m/z 1092, 970 and 282 confirm the presence of SdA epitope. **b)** MS/MS spectrum of molecular ion at m/z 1461 from sample P31. The fragments at m/z 759, and 725, 620 and 489 confirm the presence of a mixture of structures carrying the second NeuAc residue either as a sequential NeuAc or on the GlcNAc. **c)** MS/MS spectrum of molecular ion at m/z 1724 from sample P36. Fragments at m/z 646, 847, 899 and 472 confirm the presence of internal Lewis epitope.


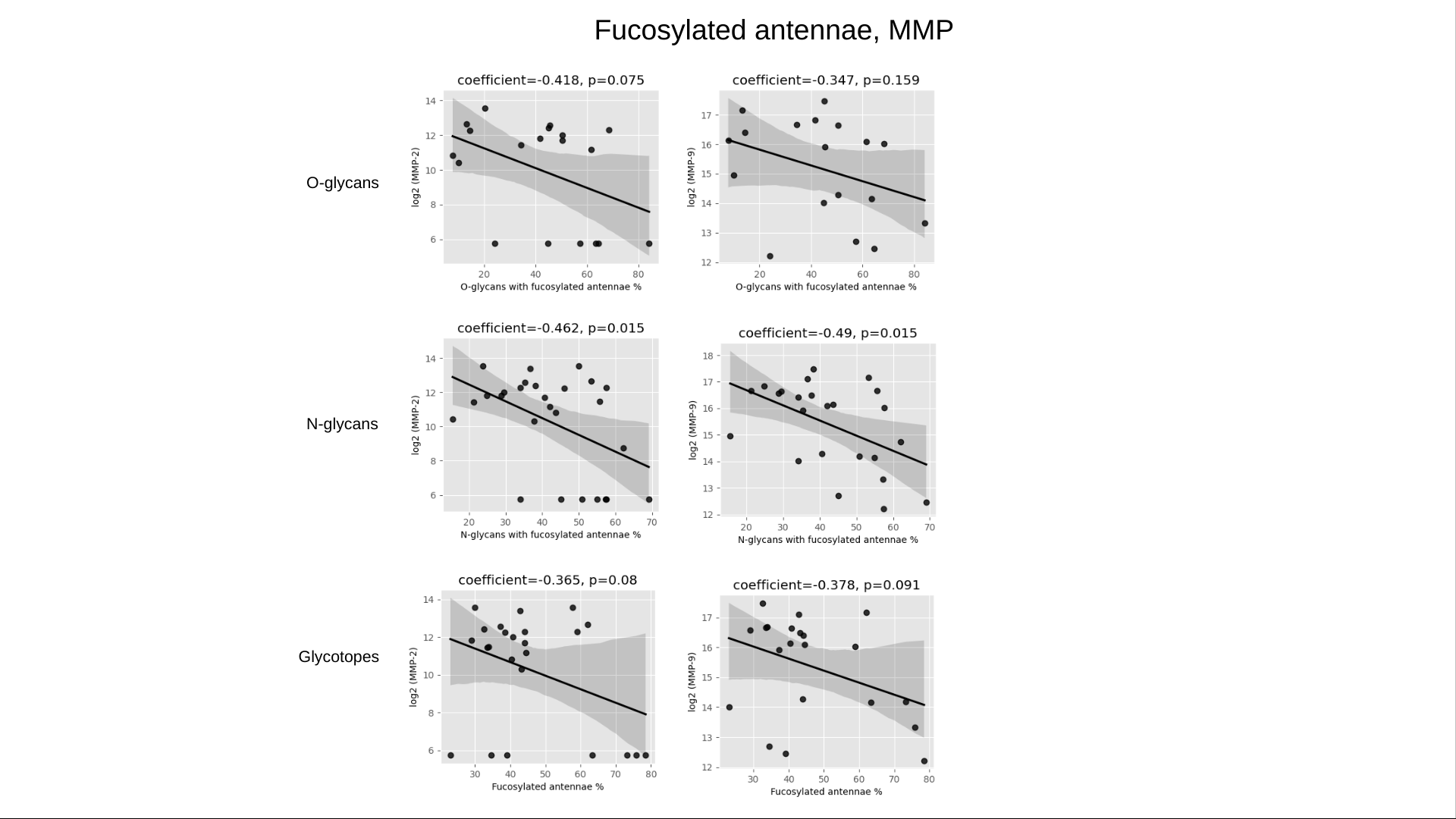


Supplementary Fig. S6. Correlation of glycans with fucosylated antennae to MMP-2 and MMP-9


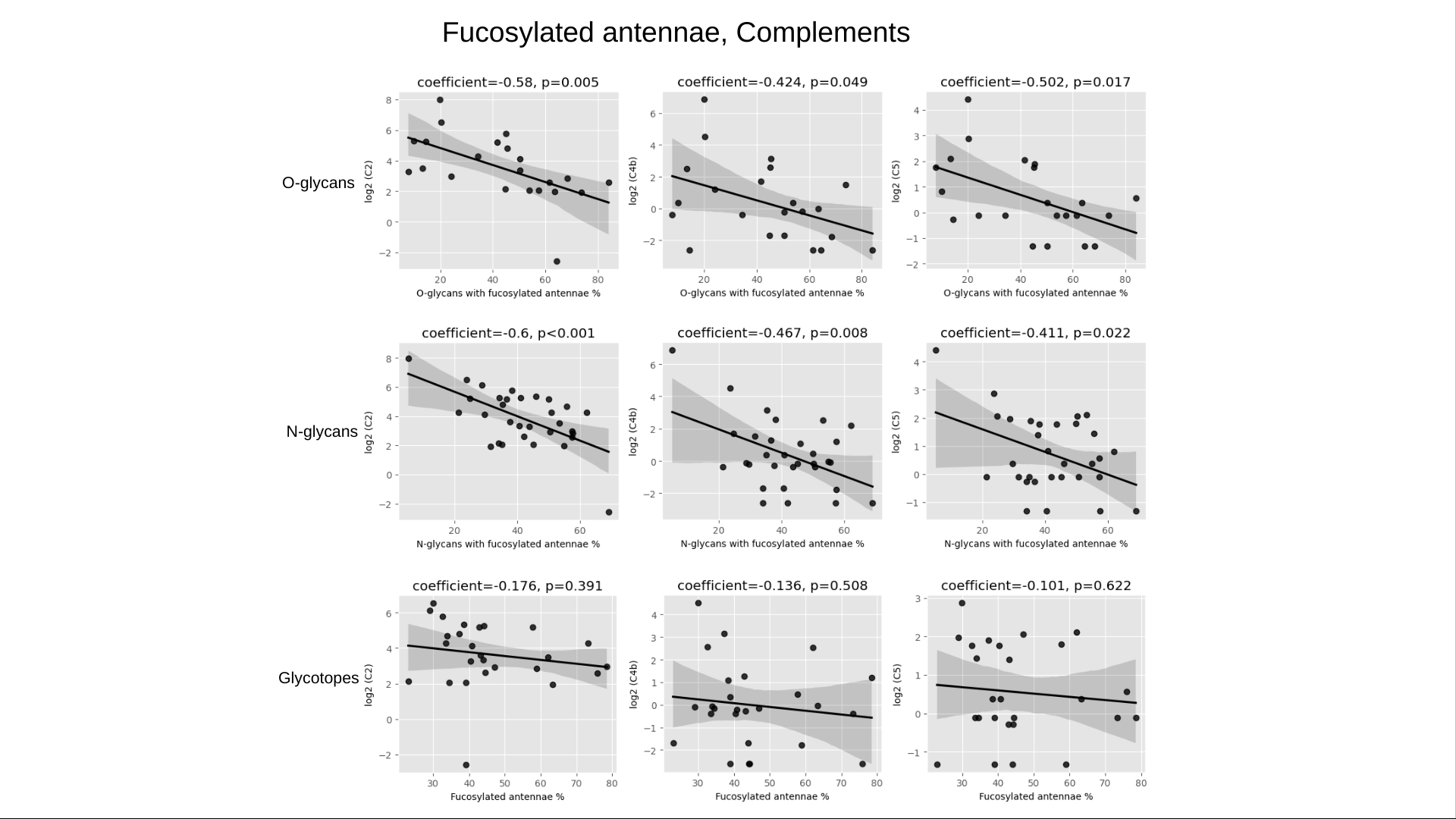


Supplementary Fig. S7. Correlation of glycans with fucosylated antennae to C2, C4b and C5.


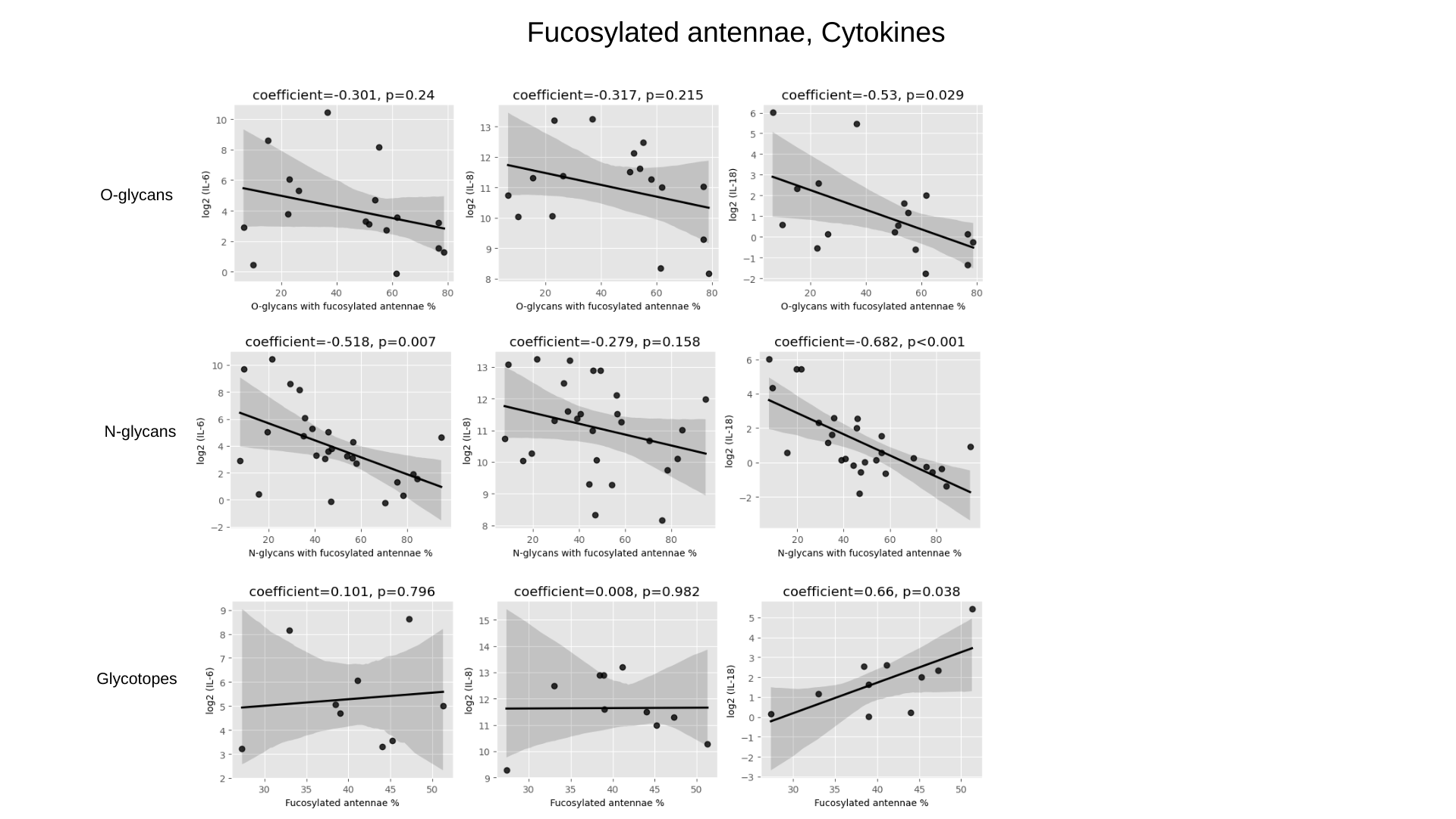


Supplementary Fig. S8. Correlation of glycans with fucosylated antennae to IL-6, IL-8 and IL-18.


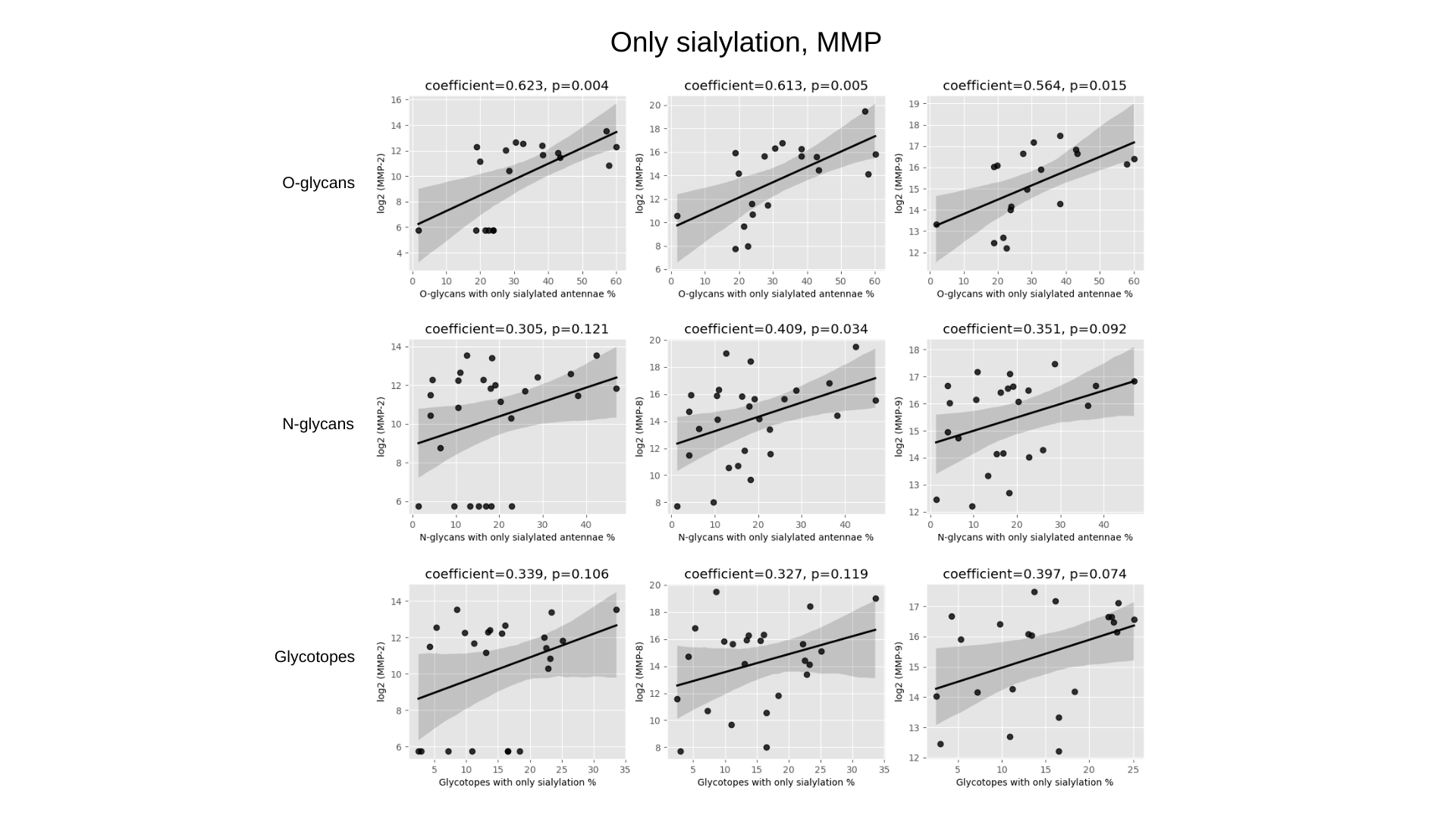


Supplementary Fig. S9. Correlation of glycans with only sialylated antennae to MMP-2, MMP-8 and MMP-9.


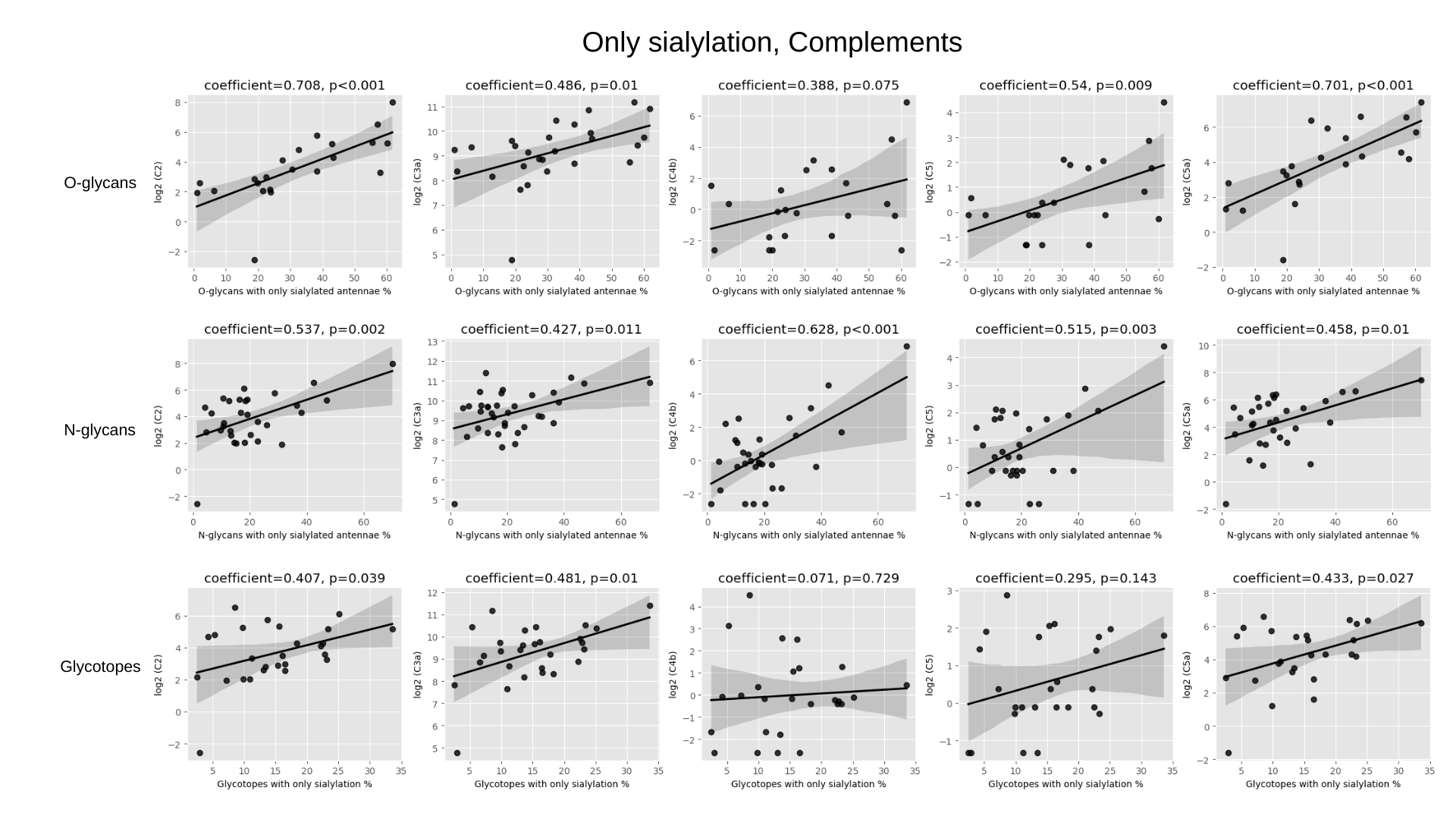


Supplementary Fig. S10. Correlation of glycans with only sialylated antennae to C2, C3a, C4b, C5 and C5a.


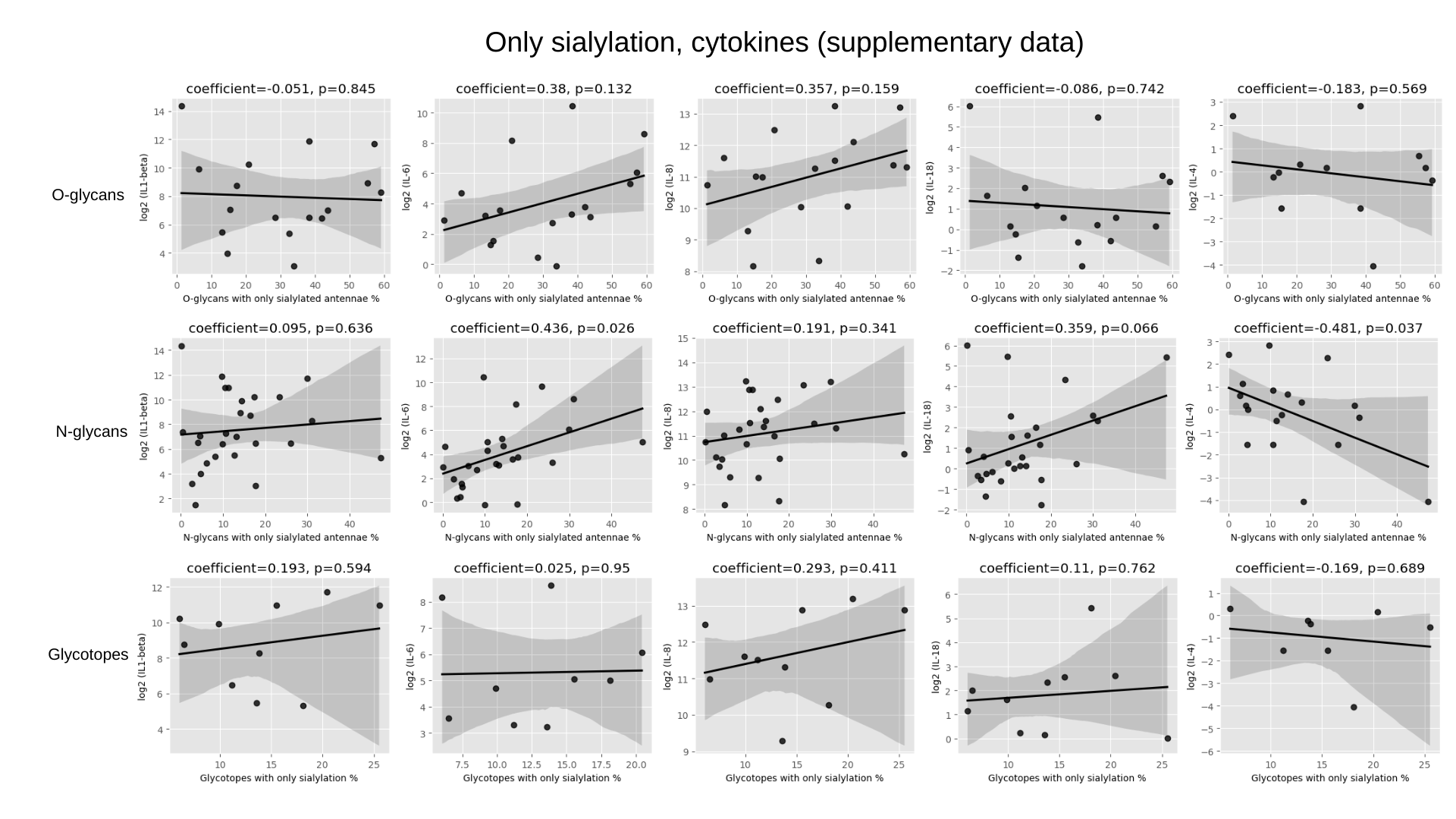


Supplementary Fig. S11. Correlation of glycans with only sialylated antennae to IL-1β, IL-6, IL-8 and IL-18.


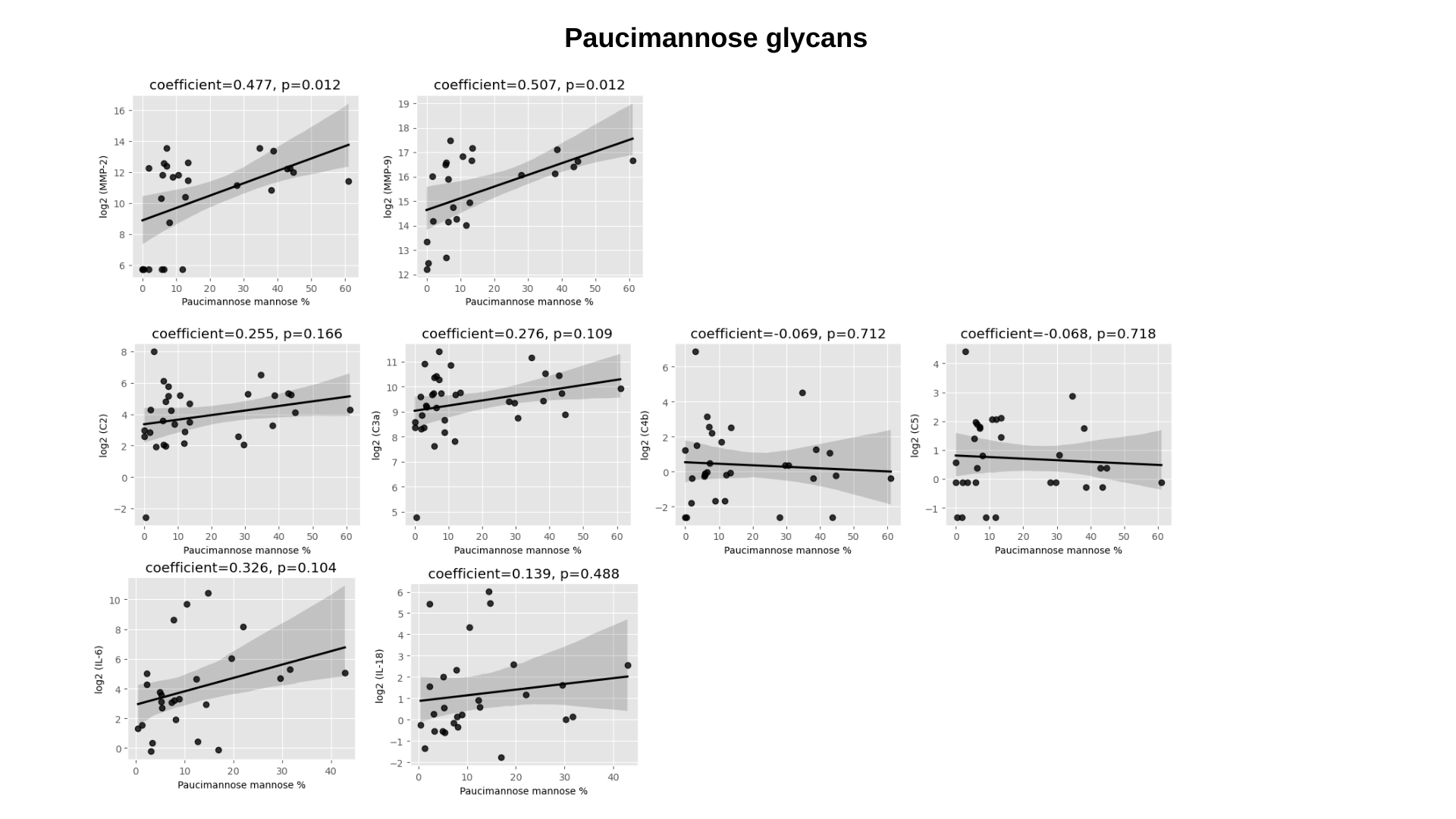


Supplementary Fig. S12. Correlation of paucimannose glycans to MMP-2, MMP-9, C2, C3a, C4b, C5, IL-6 and IL-18. Paucimannose glycans % was calculated as the summed intensity of paucimannose glycans relative to the total intensity of paucimannose and high mannose glycans.


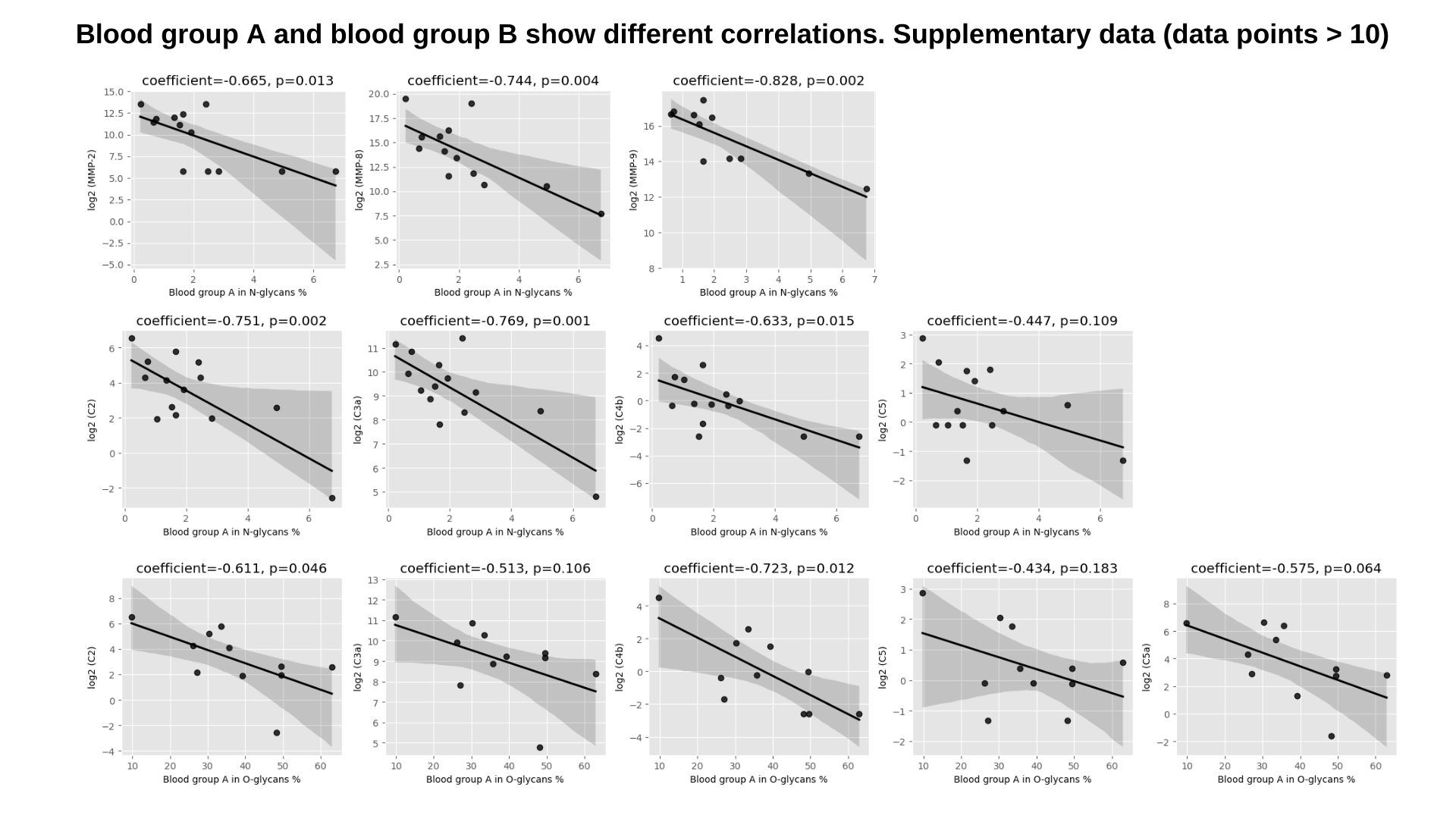


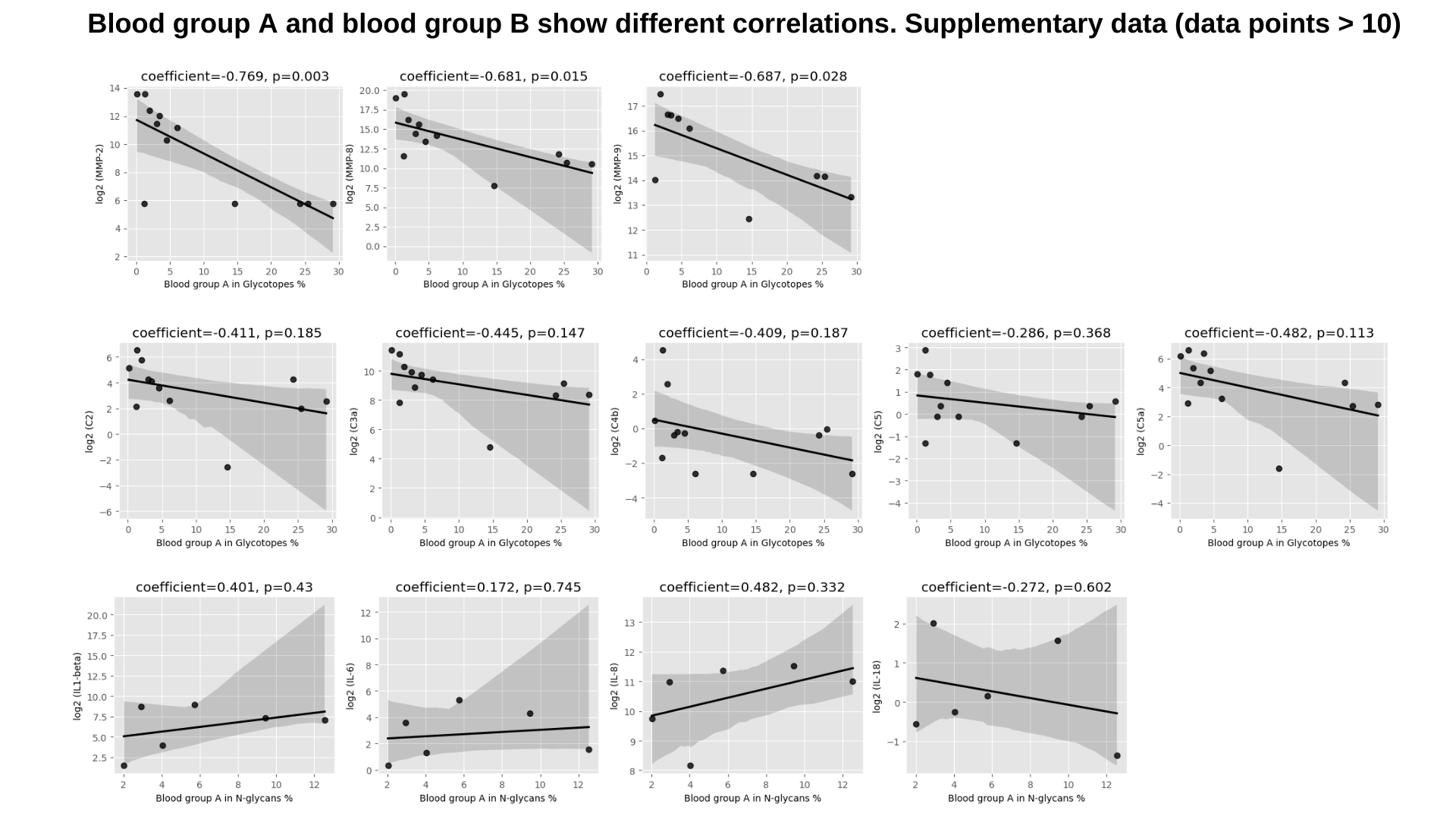


Supplementary Fig. S13. Correlation of blood group A levels to immune factors. Blood group A % in O-glycans is calculated as the summed intensity of blood group A on O-glycans in the total intensity of O-glycans in the spectrum. Blood group A in N-glycans % is calculated as the summed intensity of blood group A N-glycans in the total intensity of complex N-glycans in the spectrum.


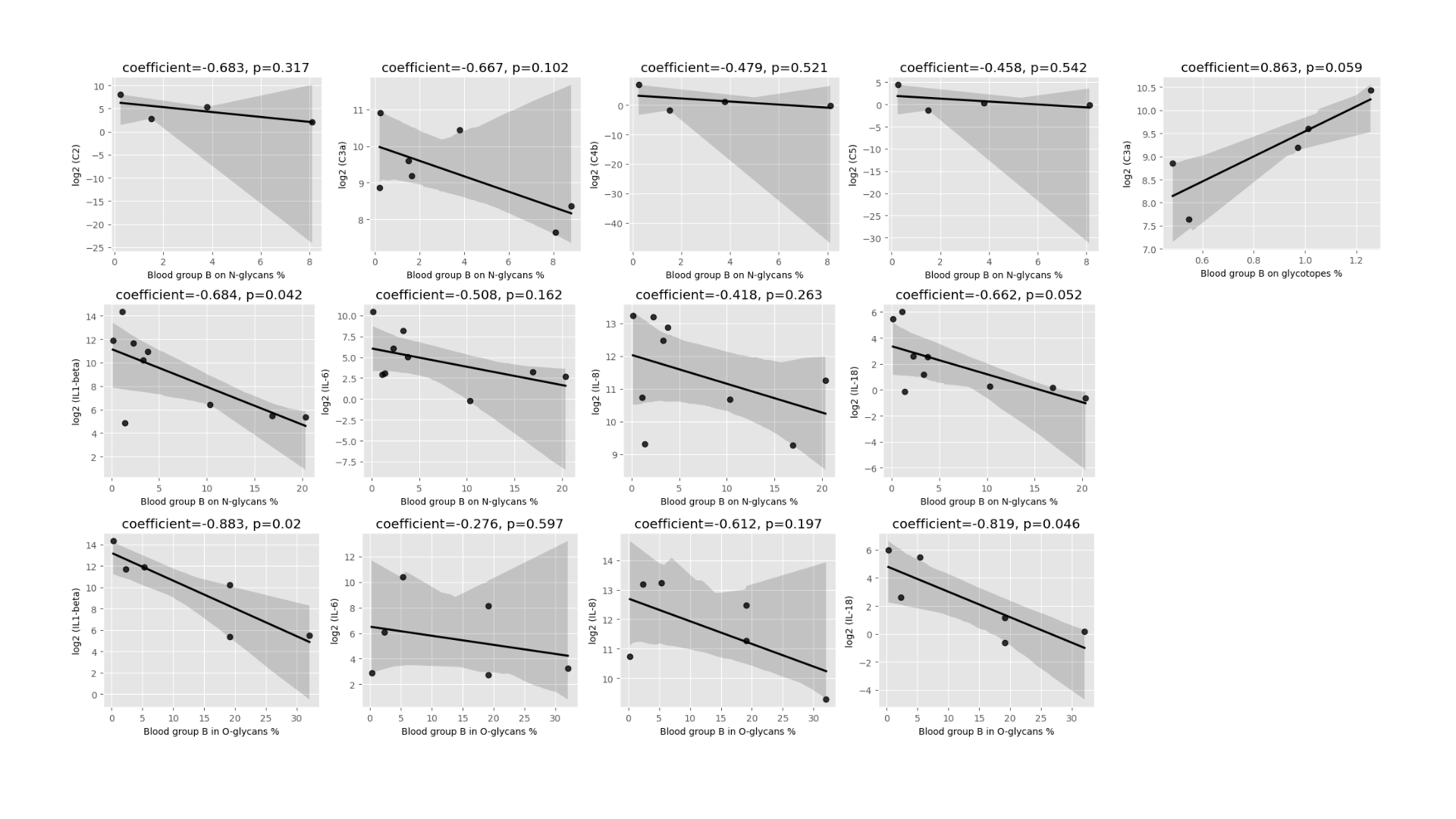


Supplementary Fig. S14. Correlation of blood group B levels to immune factors. Blood group B % in O-glycans or glycotopes is calculated as the summed intensity of blood group B on O-glycans or glycotopes in the total intensity of O-glycans or glycotopes respectively in the spectrum. Blood group B in N-glycans % is calculated as the summed intensity of blood group B N-glycans in the total intensity of complex N-glycans in the spectrum.

**a)**
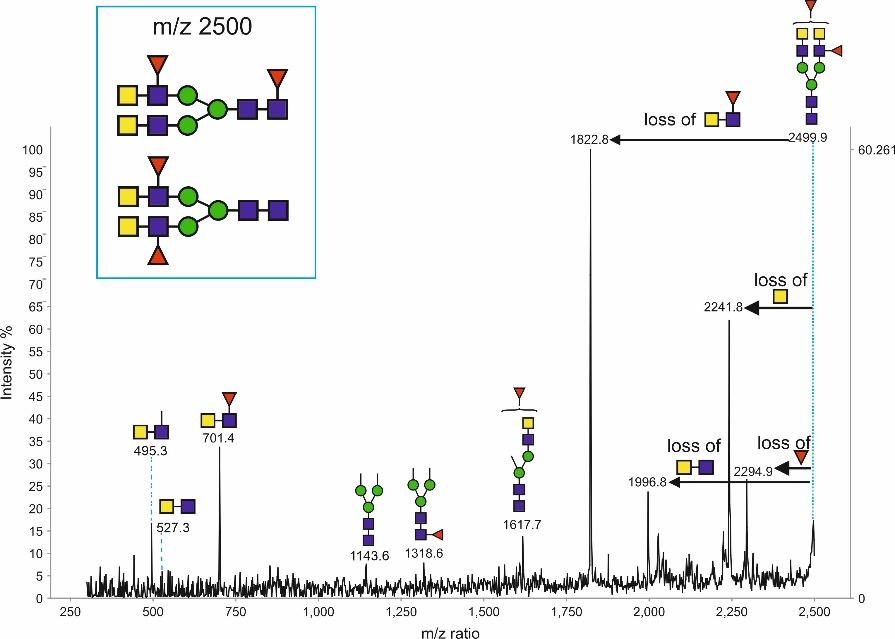


**b)**
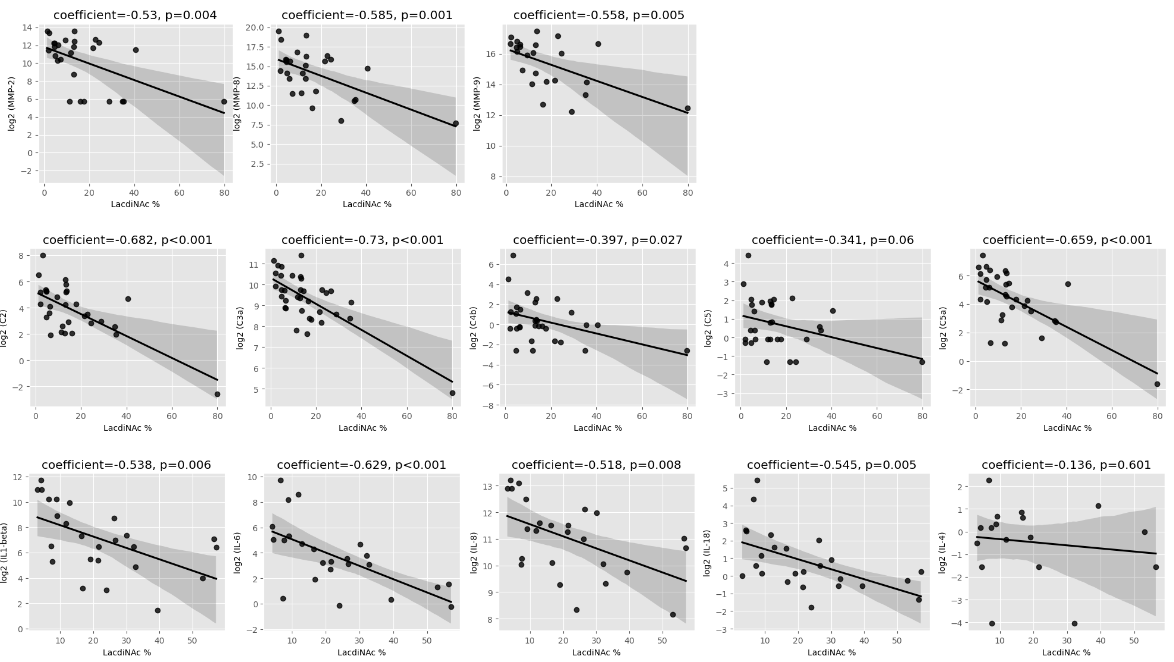


Supplementary Fig. S15. **a)** MALDI MS/MS analysis of the molecular ion at m/z 2500, from sample P29. The fragments at m/z 527, 701, 1821 and 1996 confirm the presence of LacdiNAc epitope. **b)** Correlation of LacdiNAc glycans to MMPs, Complements and cytokines (bottom panel). LacdiNAc % was calculated as the summed intensities of LacdiNAc glycans relative to the total intensities of LacdiNAc glycans, only sialylated glycans and paucimannose glycans.


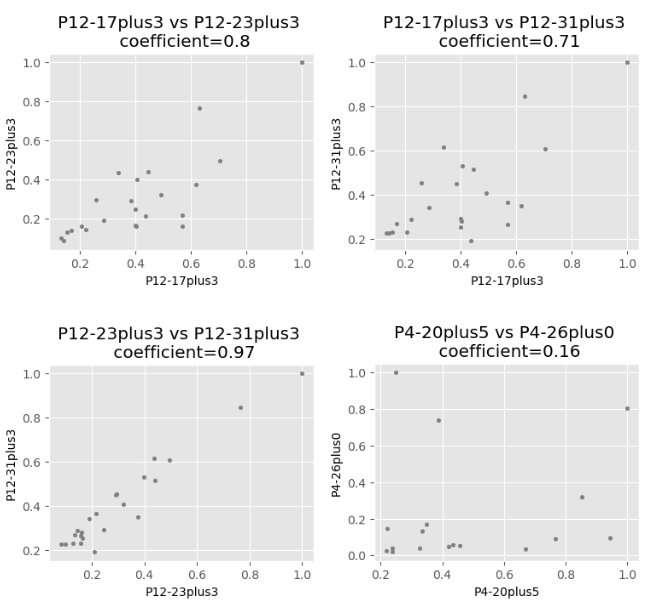


Supplementary Fig. S16. Measurement of the similarity of longitudinal glycan profiles from the term donor P12 and the preterm donor P4 by Pearson correlation analysis. The spectra from each donor were paired and the similarity between each pair was measured. Each spectrum at a mass range 1500-3500 was used for data analysis. The relative intensity of each glycan to the most abundant glycan was calculated. Glycans with relative intensity above 0.2 (20%) were used for correlation analysis.


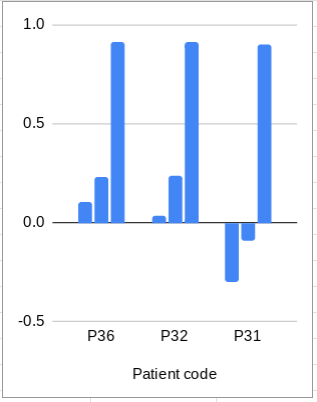


Supplementary Fig. S17. Pearson correlation coefficient values of three term pregnancies that were different from the majorities. There are three sampling time points for each donor and therefore has three values/donor.


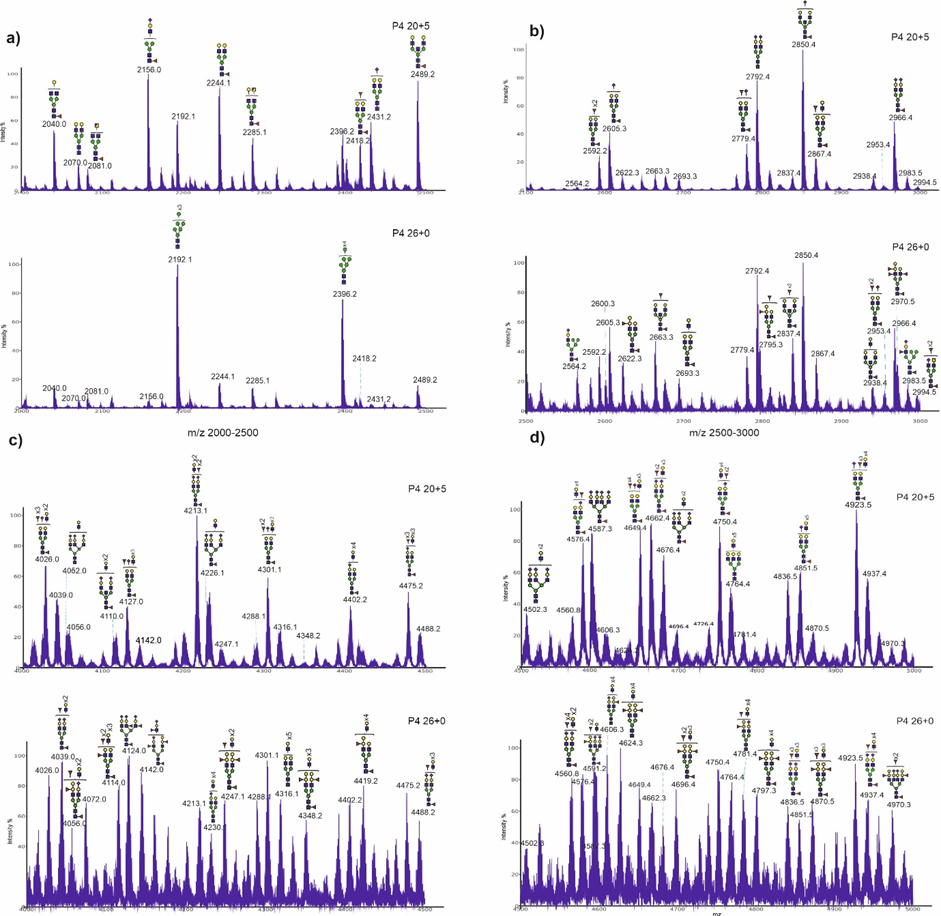


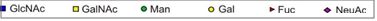


Supplementary Fig. S18. Longitudinal change of N-glycosylation of sample P4 at 20w5d (upper panel) and 26w0d (lower panel), zoomed in sections are reported in panels **a),** m/z 2000-2500, and **b),** m/z 2500-3000, **c)** m/z 4000-4500, **d)** m/z 4500/5000. Cartoon structures of main peaks are depicted in each panel. Composition is based on MALDI-MS and MS-MS analysis.


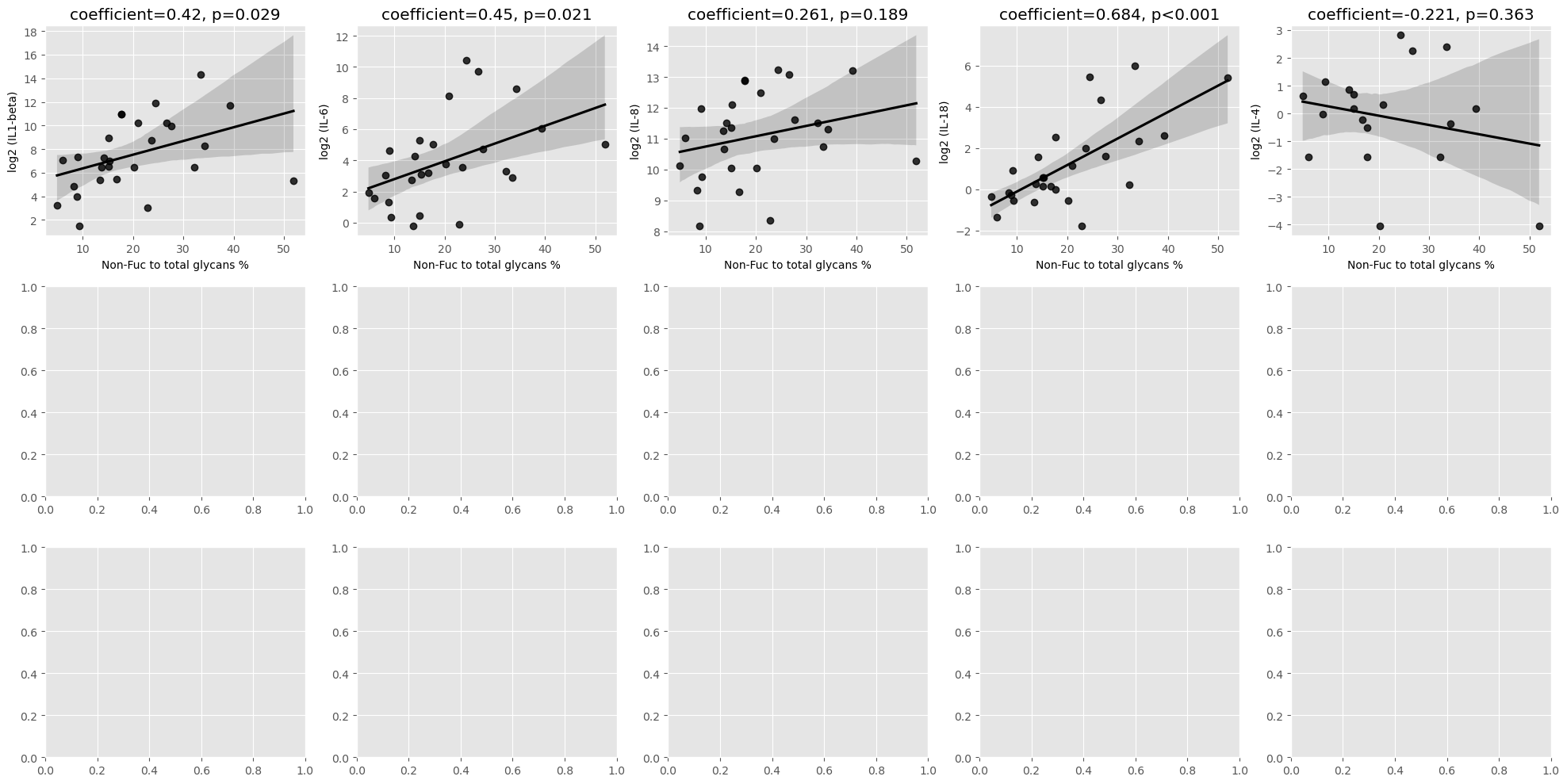


Supplementary Fig. S19. Correlation of non-fucosylated N-glycans to total glycans with 2 LacNAc units.


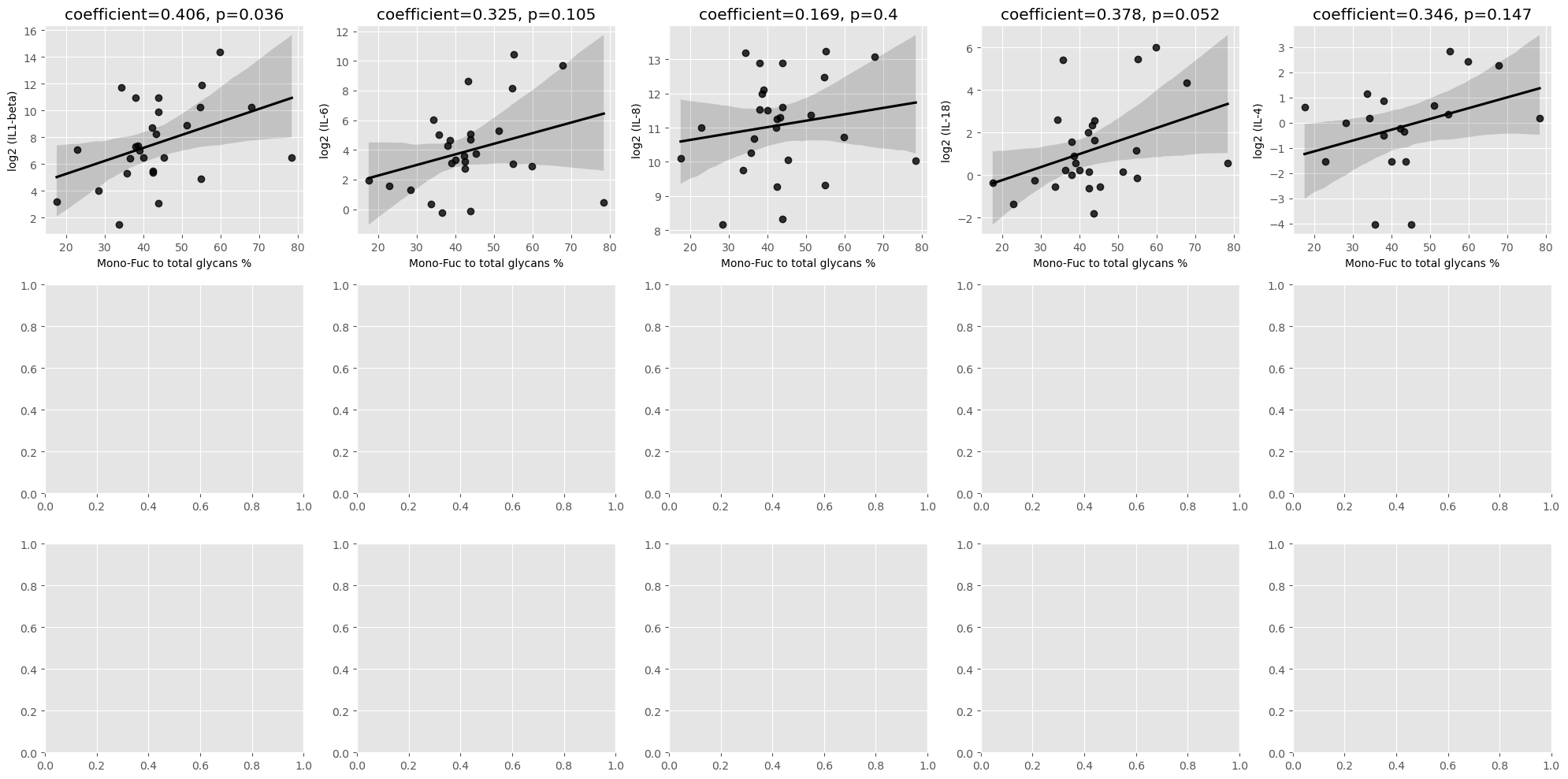


Supplementary Fig. S20. Correlation of mono-fucosylated N-glycans to total glycans with 2 LacNAc units.


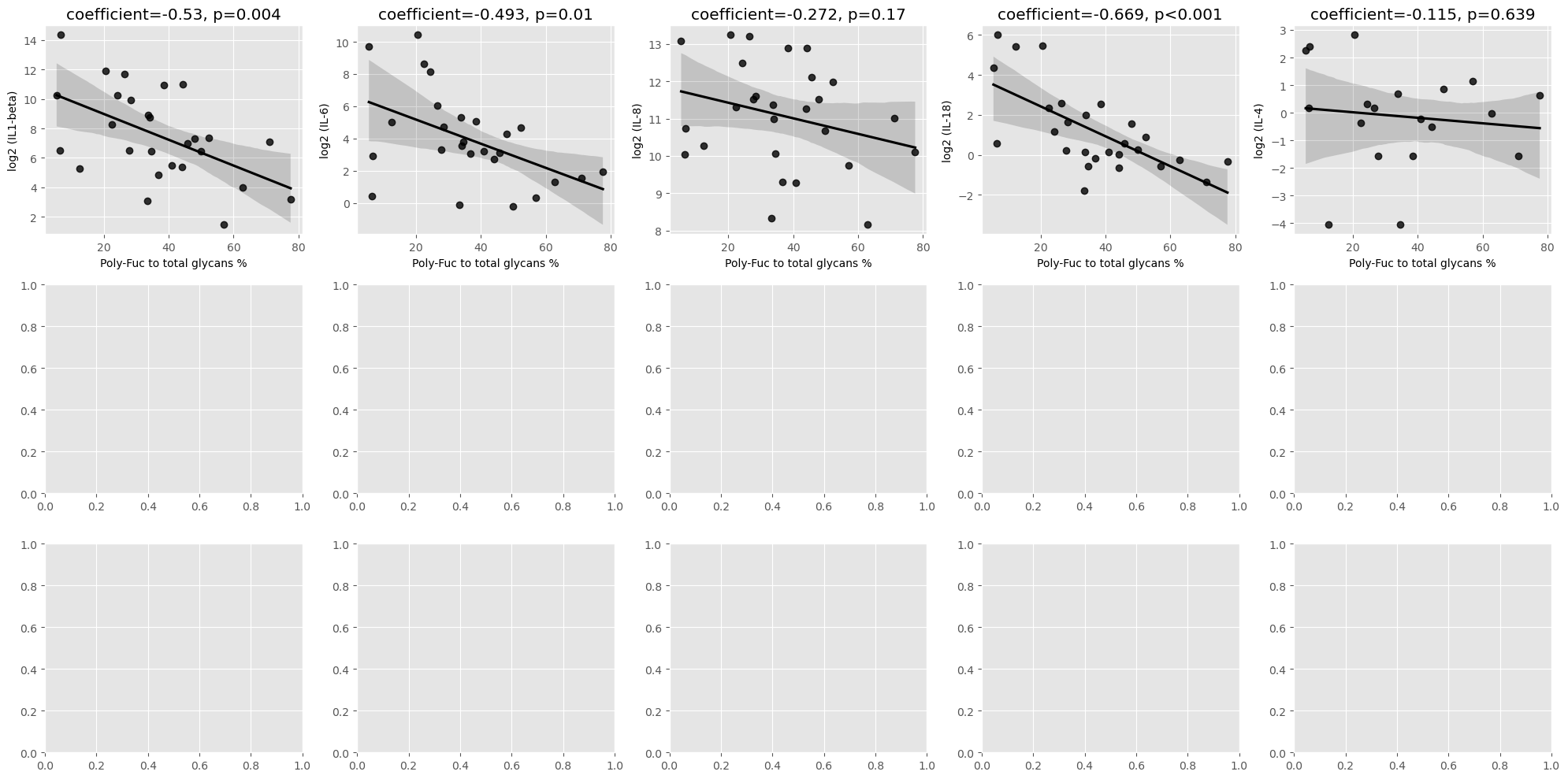


Supplementary Fig. S21. Correlation of poly-fucosylated N-glycans to total glycans with 2 LacNAc units.


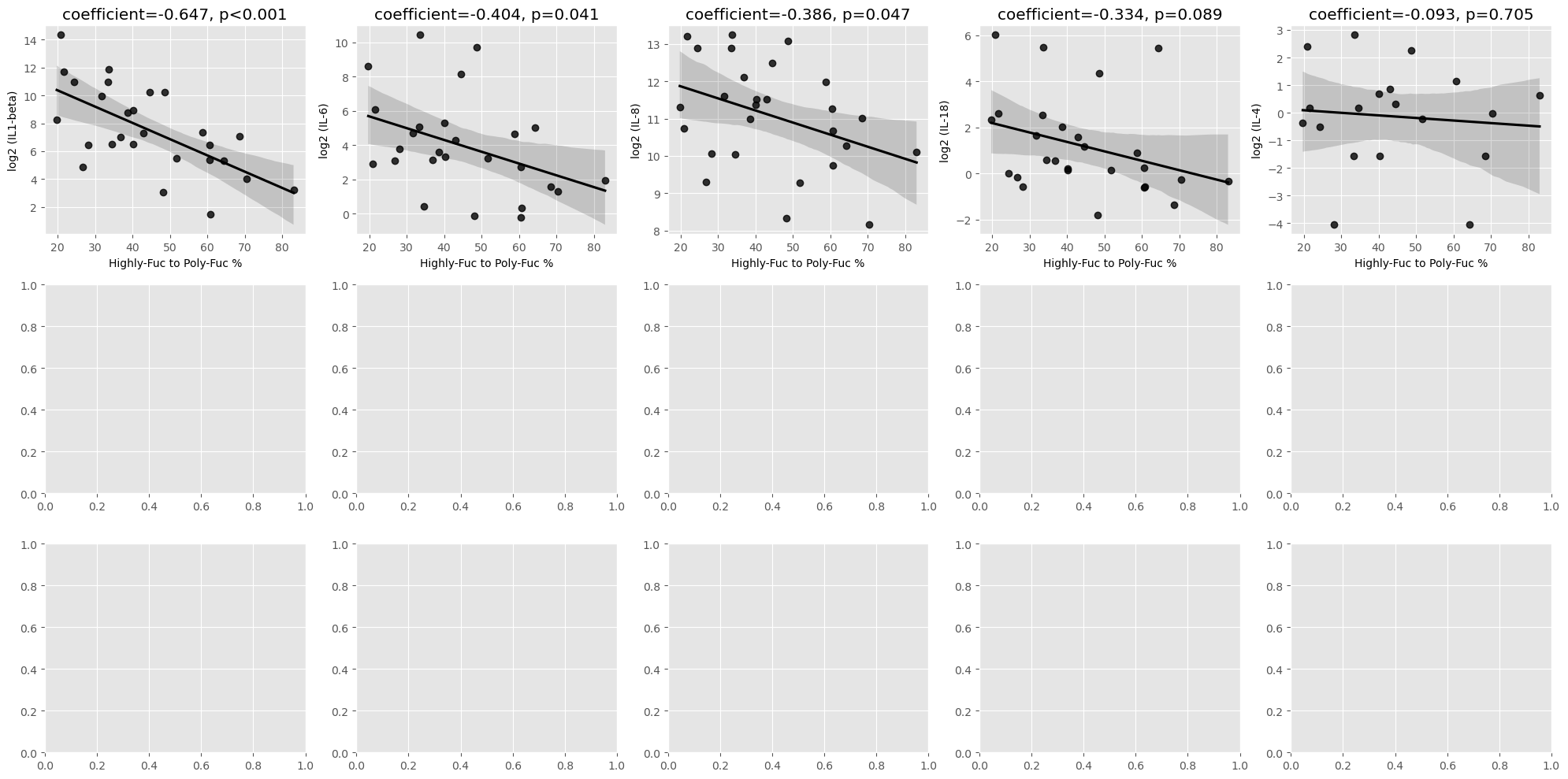


Supplementary Fig. S22. Correlation of highly-fucosylated N-glycans to poly glycans with 2 LacNAc units.


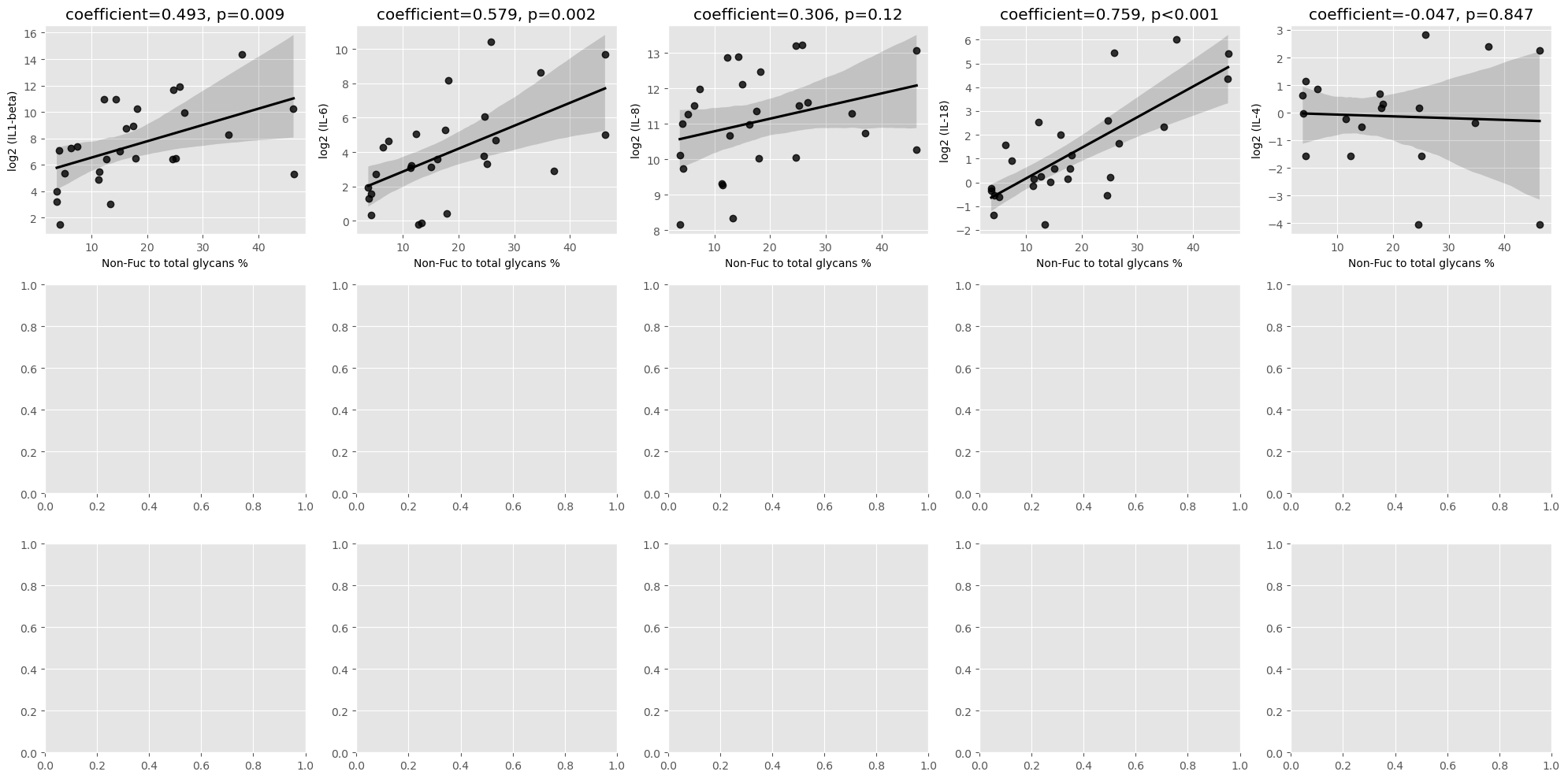


Supplementary Fig. S23. Correlation of non-fucosylated N-glycans to total glycans with 3 LacNAc units.


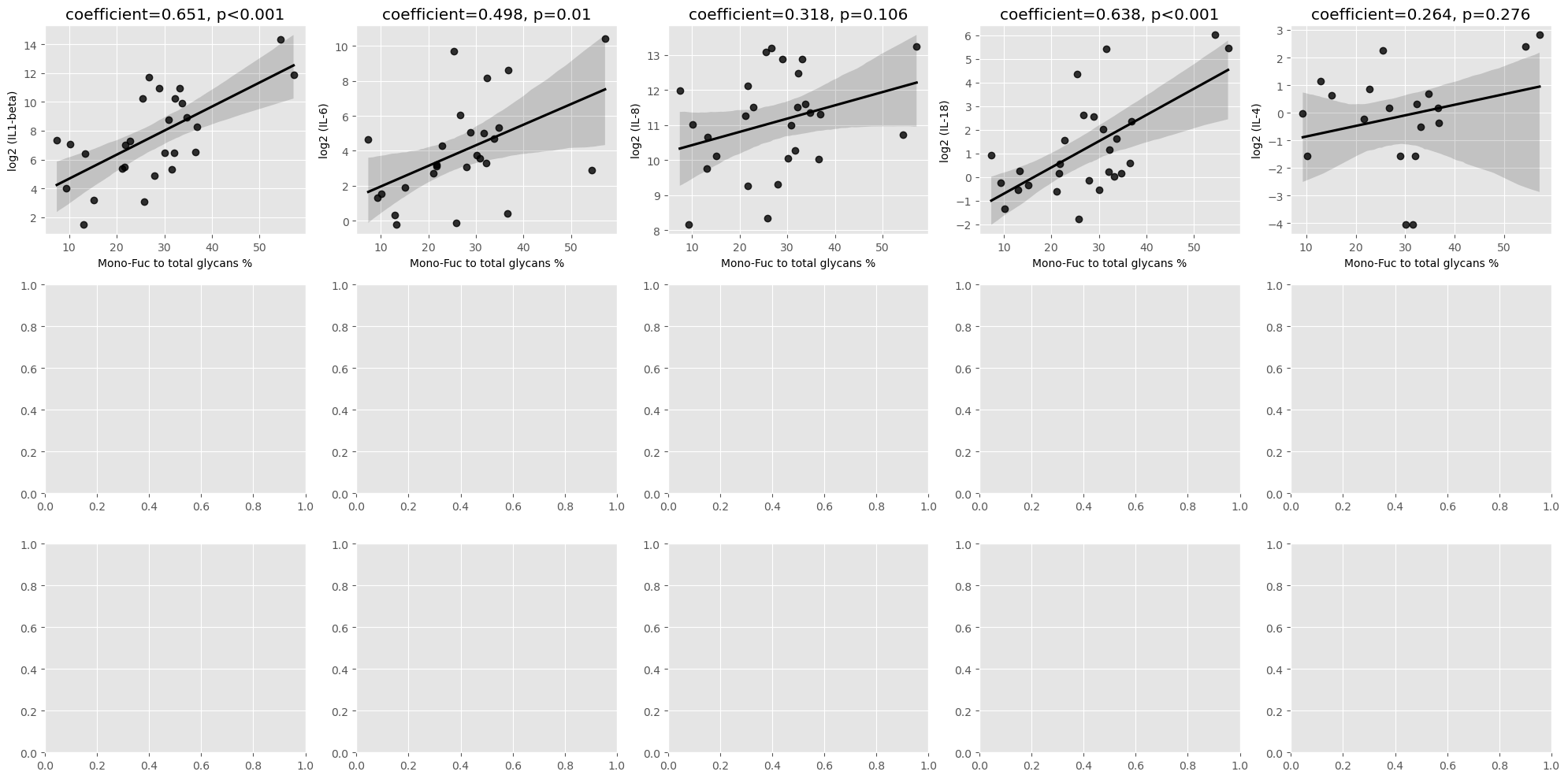


Supplementary Fig. S24. Correlation of mono-fucosylated N-glycans to total glycans with 3 LacNAc units.


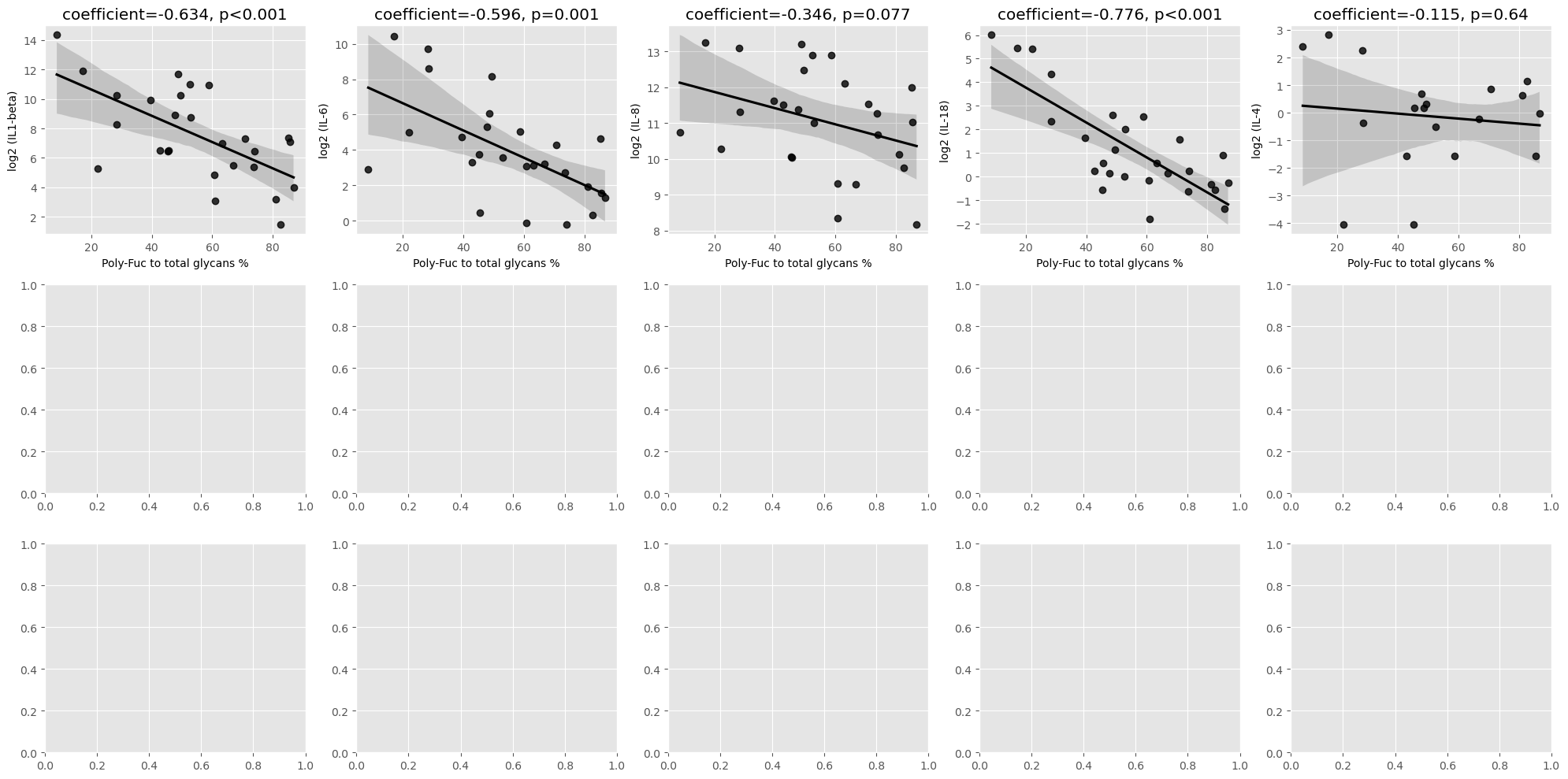


Supplementary Fig. S25. Correlation of poly-fucosylated N-glycans to total glycans with 3 LacNAc units.


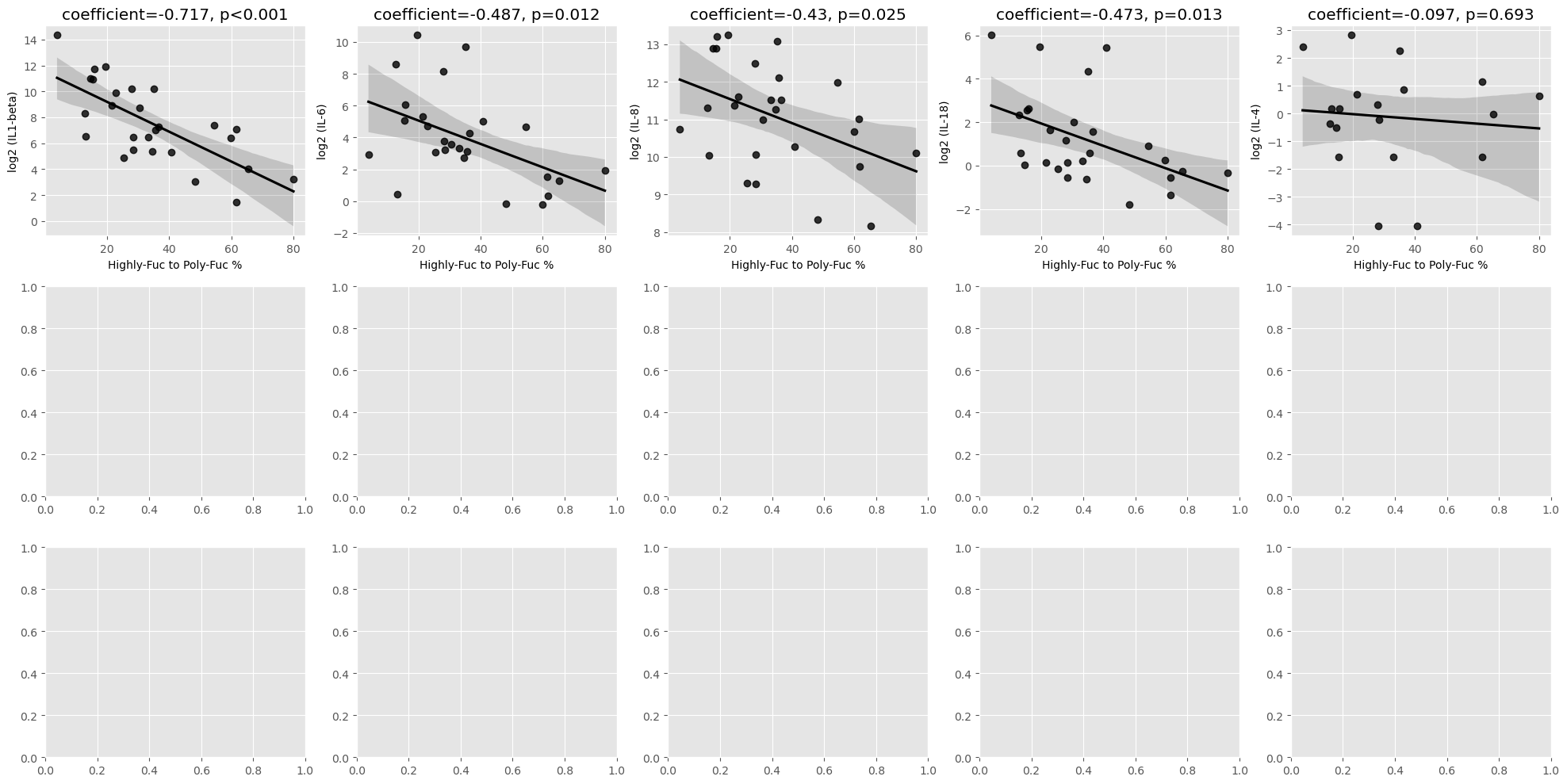


Supplementary Fig. S26. Correlation of highly-fucosylated N-glycans to poly glycans with 3 LacNAc units.


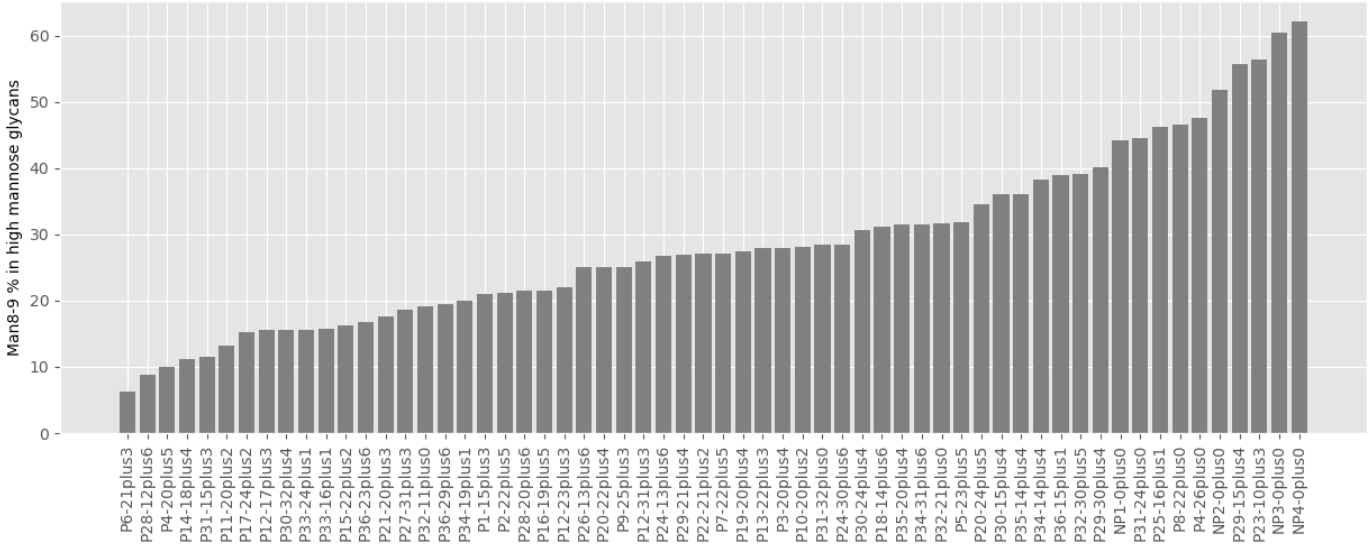


Supplementary Fig. S27. Man8-9 % in high mannose glycans. Man8-9 % is calculated as summed intensities of Man8 and Man9 relative to summed intensities of all high mannose glycans.


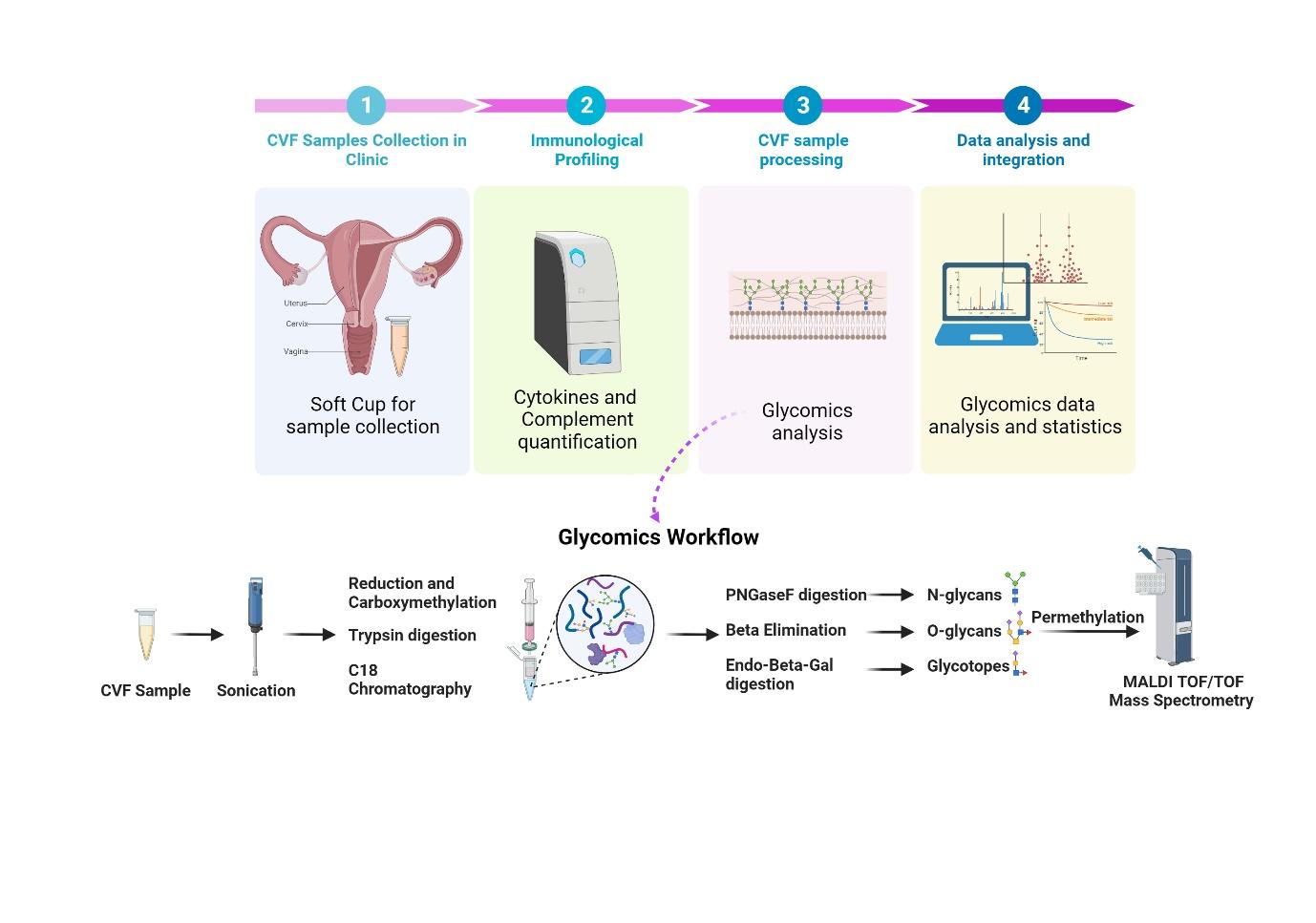


Supplementary Fig. S28. Workflow of glycomic sample processing and data acquisition. The CVF samples were lysed, reduced and carboxymethylated, digested to glycopeptides and cleaned by C18 chromatography. N-glycans were released using PNGase F and O-glycans by reductive elimination. Glycotopes on extended antennae of N- and O-glycans were released from glycopeptides using endo-beta-galactosidase. The released glycans were permethylated and analyzed by mass spectrometry. Parallel experiments were done to measure cytokines, Complements and MMPs. Figure created in **Biorender.**com.


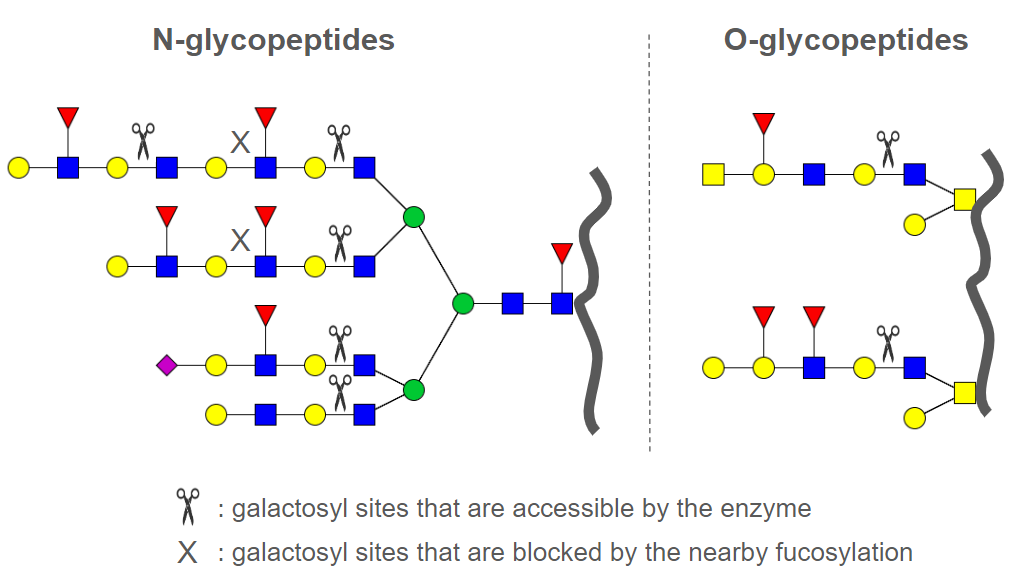


Supplementary Fig S29. Releasing of glycotopes from N- and O-glycopeptides by endo-beta-galactosidase. Sites that are accessible by the enzyme and the sites that are blocked by the nearby fucosylation are shown.
